# Metabolomic analysis and bioactivities of *Arbutus unedo* leaves harvested across the seasons in different natural habitats of Sardinia (Italy)

**DOI:** 10.1186/s12870-023-04497-0

**Published:** 2023-10-13

**Authors:** Cinzia Sanna, Ilaria Chiocchio, Manuela Mandrone, Francesca Bonvicini, Giovanna A. Gentilomi, Simona Trincia, Ferruccio Poli

**Affiliations:** 1 Department of Life and Environmental Sciences, University of Cagliari, via Sant’Ignazio da Laconi 13, Cagliari, 09123 Italy; 2 Department of Pharmacy and Biotechnology, Alma Mater Studiorum, University of Bologna, Via Irnerio 42, Bologna, 40126 Italy; 3 Department of Pharmacy and Biotechnology, Alma Mater Studiorum, University of Bologna, Via Massarenti 9, Bologna, 40138 Italy; 4 Microbiology Unit, IRCCS Azienda Ospedaliero-Universitaria di Bologna, Via Massarenti 9, Bologna, 40138 Italy

**Keywords:** NMR-based metabolomics, Antioxidant activity, Antibacterial activity, *β*-arbutin, *O*-*β*-methylglucose, Phenolics

## Abstract

**Background:**

*Arbutus unedo* L. is a wild tree of Mediterranean regions used as food and in traditional medicine and important for afforestation programs. There is no detailed information available on the variation of *A. unedo* leaves metabolome across the seasons. The leaves were analyzed by Proton nuclear magnetic resonance (^1^ H NMR)-based metabolomics, comparing samples harvested across the seasons and in ten different natural habitats of Sardinia (Italy).

**Results:**

Multivariate analysis showed the impact of seasonal variation on the metabolome: glucose and quinic acid increased in summer, while in spring sucrose was accumulated. *β*-Arbutin, the main known active principle of *A. unedo*, generally reached the highest concentration in autumn. In winter, *O*-*β*-methylglucose, γ-aminobutyric acid (GABA), flavonols (quercetin-3-*O*-*α*-rhamnoside, myricetin-3-*O*-*α*-rhamnoside, kaempferol-3-*O*-*α*-rhamnoside), catechin, and gallocatechin increased. Characteristic metabolomic features were found also for samples collected in different locations. For instance, trees growing at the highest altitude and exposed to lower temperatures produced less flavonols and catechins. The only sample collected on trees growing on limestones, dolomites, and dolomitic limestones type of soil showed generally the highest content of arbutin. The highest phenolics content was found during spring, while samples collected on flowering branches in winter were the ones with the highest flavonoid content. The antioxidant activity was also variated, ranging from 1.3 to 10.1 mg of Trolox equivalents (TE)/mL of extract, and it was positively correlated to both total phenolics and flavonoid content. Winter samples showed the lowest antibacterial activity, while summer and autumn ones exhibited the highest activity (IC_50_ values ranging from 17.3 to 42.3 µg/mL against *Staphylococcal* species).

**Conclusion:**

This work provides ^1^ H-NMR fingerprinting of *A. unedo* leaves, elucidating the main metabolites and their variations during seasons. On the basis of arbutin content, autumn could be considered the balsamic period of this taxon. Samples collected in this season were also the most active ones as antibacterial. Moreover, an interesting metabolomic profile enriched in catechins and flavonols was observed in leaves collected in winter on flowering branches which were endowed with high antioxidant potential.

**Supplementary Information:**

The online version contains supplementary material available at 10.1186/s12870-023-04497-0.

## Background

*Arbutus unedo* L. (Ericaceae family), also known as strawberry tree, is an evergreen shrub or small tree native to the Mediterranean region and Western Europe. It grows on a wide variety of soil types, and it is strongly versatile, dry-adapted, and fire resistant. For these peculiar features, *A. unedo* is recently gaining importance for afforestation programs, to intensify forest discontinuity, especially in southern Europe [1].

*A. unedo* is generally regarded as a wild edible plant with high nutritional value. It is characterized by the production, during the winter season, of both pinkish-white flowers and red fruits, the latter maturing in about 12 months, from the flowers of the previous year. Its fruits are typically eaten raw or used to prepare jam and alcoholic beverages, as well as used for animal feeding [2, 3]. Some countries have implemented programs aimed at selecting *A. unedo* genotypes with high fruit quality, promoting extensive cultivation, and preventing deforestation and excessive harvesting [4].

Fruits and leaves of this plant are used in the traditional medicine of Iberian Peninsula and Sardinia (Italy) to treat a wide number of illnesses [2, 5, 6]. In support to the ethnobotanical uses and behind this, several studies focused on the biological activity of *A. unedo* have been published (many of them have been extensively reviewed by Morgado et al. [2]), demonstrating a number of health-promoting properties of this plant and its metabolites.

*A. unedo* leaves contain a significantly higher content of polyphenols than fruits; moreover, arbutin, an important bioactive compound present in this plant, was found only in leaves [4]. In addition, *A. unedo* leaves have uroantiseptic, diuretic, and astringent properties and are used in folk medicine for treating inflammation, hypertension, and diabetes [7]. In Sardinian traditional medicine the leaves are prepared as a decoction and used as febrifuge, vulnerary, and to treat intestinal pains, asthma, and bronchitis [8–10][8–10]. Taking also into account the increasing demand for food that have a positive impact on health, the leaf of this plant can be valuable as functional food.

Despite the notable properties of this plant, little is known about the environmental influences on its metabolome which is relevant in the investigation of *A. unedo* biological activities as well as to implement plant breeding strategies.

The importance of *A. unedo* in several fields, ranging from food and medicine to ecology, inspired us to deepen the knowledge of this plant both in terms of phytochemical profile and bioactivities.

In particular, we employed ^1^ H NMR-based metabolomics approach to follow the impact of seasonality on the metabolome of *A. unedo* leaves. Previous studies have shown the influence of the stage of plant development and climatic conditions on the concentration of secondary metabolites, however in strawberry tree, no relationship was found between the metabolites analyzed and a specific time of year [11]. Metabolomics already proved to be a valid tool to tackle the phytochemical complexity of plant matrices facilitating the extraction of information by the comparison of a high number of samples and offering the possibility to handle the data through multivariate data treatment [12, 13]. Moreover, this approach aids databases creation, together with the reuse and recycling of data.

*A. unedo* leaves were harvested across the four seasons, from trees growing in ten different natural habitats of Sardinia (Italy), an island with a high level of biodiversity. Samples were subjected to ^1^ H NMR profiling followed by multivariate data treatment with the purpose of investigating the influence of environment and season on leaves metabolome. In addition, differences between leaves collected in winter on fruiting branches and on flowering branches were explored for the first time. *A. unedo* leaves were already proved to be a promising source of antioxidant and antibacterial molecules [14, 15], however, the comparison between samples collected across the seasons and in different habitats was never performed. For this reason, in this work the total phenolic and flavonoid content, the in vitro antioxidant and antibacterial activities towards a panel of Gram positive (*S. aureus*, *S. epidermidis*, *E. faecalis*) and Gram negative bacteria (*E. coli*, *K. pneumoniae*), together with the cytotoxicity of the samples, were assayed and discussed.

## Results

### ^**1**^** H NMR based metabolomic analysis**

In order to compare the metabolomic profiles of the samples, unsupervised Principal Component Analysis (PCA) and then supervised Partial Least Squares Discriminant Analysis (PLS-DA) multivariate data analyses were performed, using as *x* variables the bucketed ^1^ H NMR spectra.

According to the PCA (Fig. S1) the metabolomic profile of *A. unedo* leaves varies across the seasons. Moreover, slight metabolomic differences were found between the leaves harvested during winter (W), from flowering branches (W_FL_) and fruiting (W_FR_) branches.

**Fig. 1:**
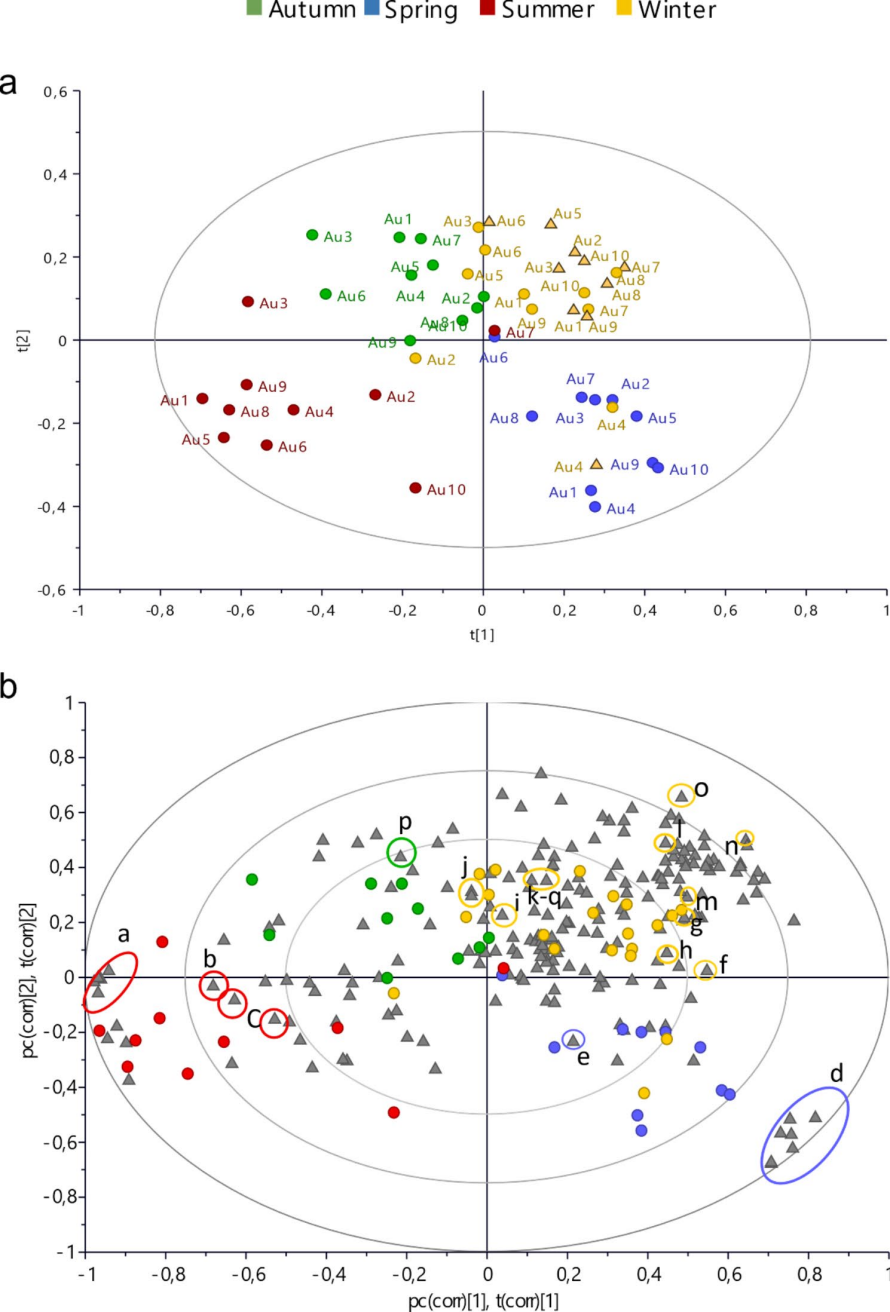
^1^ H NMR-based PLS-DA of *A. unedo* (Au) leaves. Seasons were used as discriminant classes. **a** Score scatter plot of the model, samples harvested in ten diverse locations are indicated by numbers, and different seasons are indicated by the different colors, leaves of winter fruiting branches (W_FR_) are represented by the yellow triangles **b** Biplot of the model, where the influence of bucketed ^1^ H-NMR signals (grey triangles) on samples distribution in the score scatter plot is represented. Letters indicate the diagnostic NMR signals of the most varied metabolites: a = quinic acid, b = *α*-glucose, c = *β*-glucose, d = sucrose e = gallotannin (δ 7.15), f = formic acid, g = alanine, h = myricetin-3-*O*-α-rhamnoside, i = quercetin-3-*O*-α-ramnhoside, j = afzelin, k = catechin, l = threonine, m = GABA, n = malic acid, o = 3-*O*-β-methylglucose, p = arbutin, q = gallocatechin

In order to easily associate spectral signals and seasonal variations, a supervised PLS-DA model was built, using the seasons of harvesting as discriminant classes (Fig. 1). The goodness of fit was given by *R*^*2*^*y*(cum) = 67.5%, and *R*^*2*^*x*(cum) = 73.4%, while the goodness of prediction *Q*^*2*^(cum) was 56.3%. The permutation test gave *Q*^*2*^(cum) = 55.9% and intercept on *y* axis of *Q*-line was 0.349, while *R*^*2*^(cum) was 74.3% and the intercept on *y* axis of *R*-line was 0.158. Significance testing of the model based on ANOVA of the cross-validated residuals (CV-ANOVA) gave *p* = 3.84 × 10^− 12^ and F = 6.2.

**Fig. 2:**
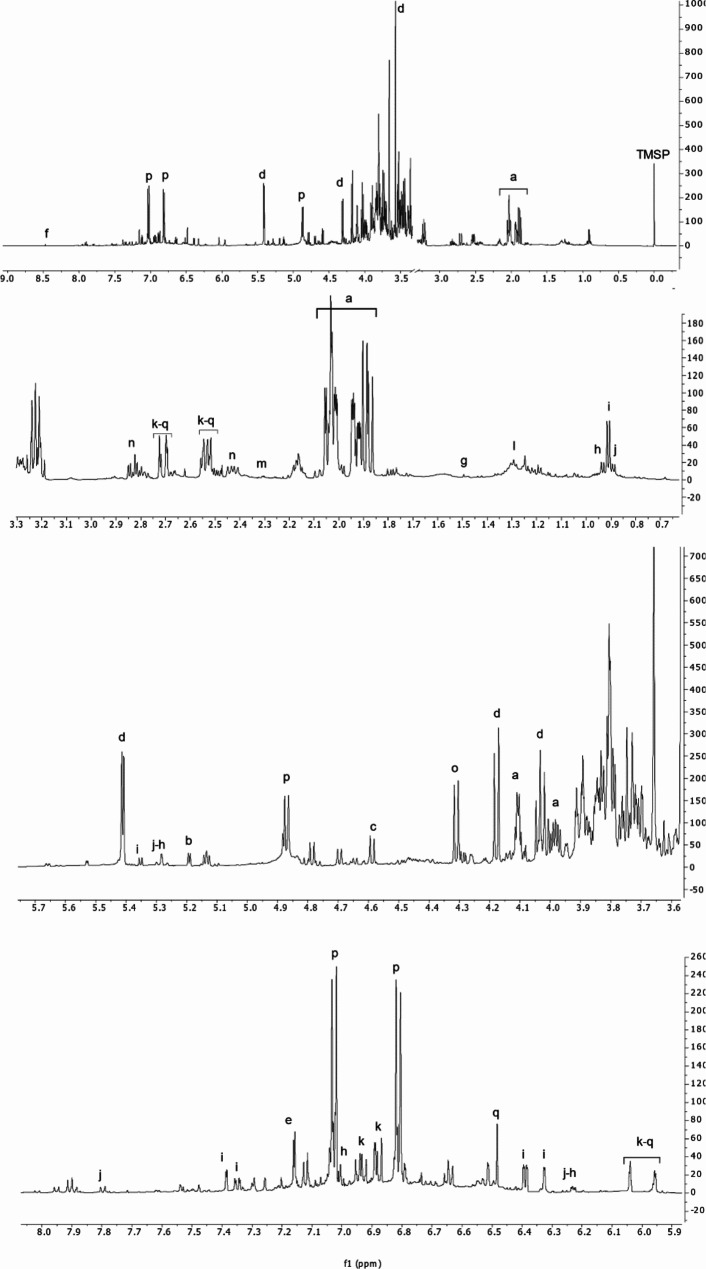
^1^ H NMR spectrum of a representative *A. unedo* sample (Au1 W_FL_). On top of the figure is shown the full spectrum followed by extended spectral regions. The metabolites important for the discrimination of different samples were identified as: a = quinic acid, b = α-glucose, c = β-glucose, d = sucrose, e = galloyl moiety, f = formic acid, g = alanine, h = myricetin-3-*O*-α-rhamnoside, i = quercetin-3-*O*-α-rhamnoside, j = afzelin, k = catechin, l = threonine, m = GABA, n = malic acid, o = 3-*O*-β-methylglucose, p = β-arbutin, q = gallocatechin

The Biplot of PLS-DA (Fig. 1B) indicated the impact of ^1^ H-NMR signals on the distribution of the samples in the score scatter plot. Through the subsequent interpretation of the spectra (Fig. 2), it was possible to establish that W was characterized by the highest amount of alanine, threonine, GABA, formic acid, malic acid, and specific flavonoids.

In order to unambiguously elucidate the structure of these flavonoids, a pre-purification procedure was carried out. The fractions containing the ^1^ H NMR signals due to flavonoids underwent further NMR and MS (Mass Spectrometry) experiments. Following this procedure, three *α*-rhamnosyl flavonols, namely quercetin-3-*O*-α-rhamnoside, myricetin-3-*O*-α-rhamnoside and afzelin, and two catechins (catechin and gallocatechin) were identified. In particular, samples collected on W_FR_ were generally less rich in these aromatic compounds than the samples from W_FL_.

The PLS-DA found also that winter (W) samples had the highest intensity of two others ^1^ H NMR signals, namely a doublet at δ 4.30 (*J* = 7.9 Hz) and a singlet at δ 3.56. The integrals ratio of these two signals was kept constant in all the samples, and it was 1:3, respectively. This suggested that they were part of the same molecule. The chemical shifts, the coupling constants, and the integrals of these two signals led us to assume that this molecule could be 3-*O*-β-methylglucose.

Spring (Sp) samples had the highest content of sucrose, and an aromatic compound (δ 7.15), which might be a gallotannin; in fact, compounds belonging to this class were already found in *A. unedo* leaves (Martins et al., 2021). To acquire more information both J-res (J-resolved Spectroscopy) and COSY (^1^ H-^1^ H homonuclear correlation spectroscopy) spectra were acquired, and this signal, resonating at δ 7.15, had no COSY correlations with other signals, resulting to be a singlet by J-res (Fig. S2), supporting the idea that this signal could be due to a gallic acid moiety. Further studies are required to fully elucidate this compound.

Summer (S) samples were characterized by an increment of glucose and quinic acid, while in autumn (A) the content of arbutin was generally increased. Through ^1^ H NMR profiling it was possible to assess that the glucose moiety of arbutin was in the β anomeric form, since the coupling constant of the doublet ascribable to the anomeric proton at δ 4.87 was 7.7 Hz.

**Fig. 3:**
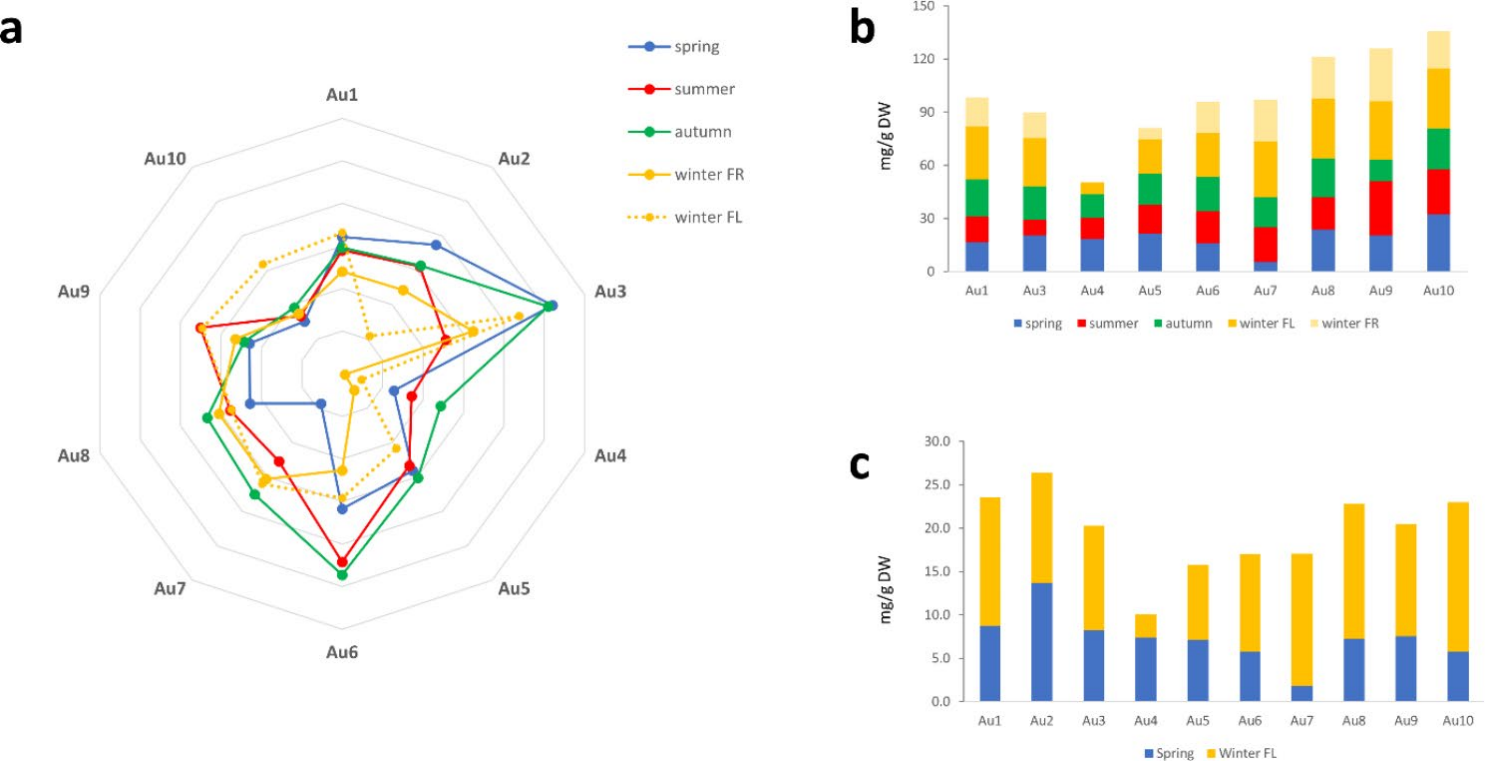
Variation across the seasons of **(a)** arbutin, **(b)** flavonoid content and **(c)** flavonols content. Flavonoid content was obtained as mean of flavonols (afzelin, quercetin-3-*O*-α-rhamnoside, myricetin-3-*O*-α-rhamnoside) and catechins (gallocatechin and catechin) content. Metabolites content is expressed in mg/g of DW and were quantified by ^1^ H NMR profiling

In addition to the influence exerted by the seasons on the metabolome of the samples, also the collection site could contribute to its variation. For instance, the content of arbutin of Au3 was one of the highest in all seasons, ranging from 52 to 23.6 mg/g DW (dry weight of plant material) (Fig. 3 and Table S2). Although the content of arbutin showed no correlations with all the factors here considered, it is noteworthy, that Au3 was the only sample collected from trees growing on calcareous soil (classified as dolomitic limestone).

Conversely, Au10 and Au4 showed a low arbutin content in all seasons, ranging from 0.7 to 31.8 mg/g DW (Fig. 3).

As showed by PLS-DA, Au4 metabolomic profile in W resulted closer to that of the other samples in Sp (Fig. 1A). Semi-quantitative analysis of the most significant compounds found by ^1^ H NMR profile was also performed as reported in Table S2. The content of flavonols and catechins found by ^1^ H NMR profiling was summed up in order to emphasize the differences between the samples, and this made once more evident the low flavonols content of Au4 in all seasons except Sp (Fig. 3B). In particular, while in all samples flavonols were increasing from Sp to W_FL_, in Au4 W_FL_ showed the lowest content of these compounds (Fig. 3C).

Notably, Au4 has been obtained from trees growing at the highest altitude (957 m asl) and was exposed to the lowest temperature in all seasons (Table S1).

Furtherly, an Orthogonal Partial Least Squares (OPLS) model was built using as *y* variable the diagnostic ^1^ H NMR signal of arbutin (*y* = intensity of the bucket at δ 6.78–6.82) (Fig. S3), highlighting an inverted trend between the concentration of arbutin and 3-*O*-rhamnosyl-flavonols. This might be due to a common precursor of these metabolites, which might preferentially undertake one pathway instead of the other. Glucose and sucrose concentration resulted also decreased when arbutin concentration increased.

### Total phenolic and flavonoid content and antioxidant activity

**Fig. 4:**
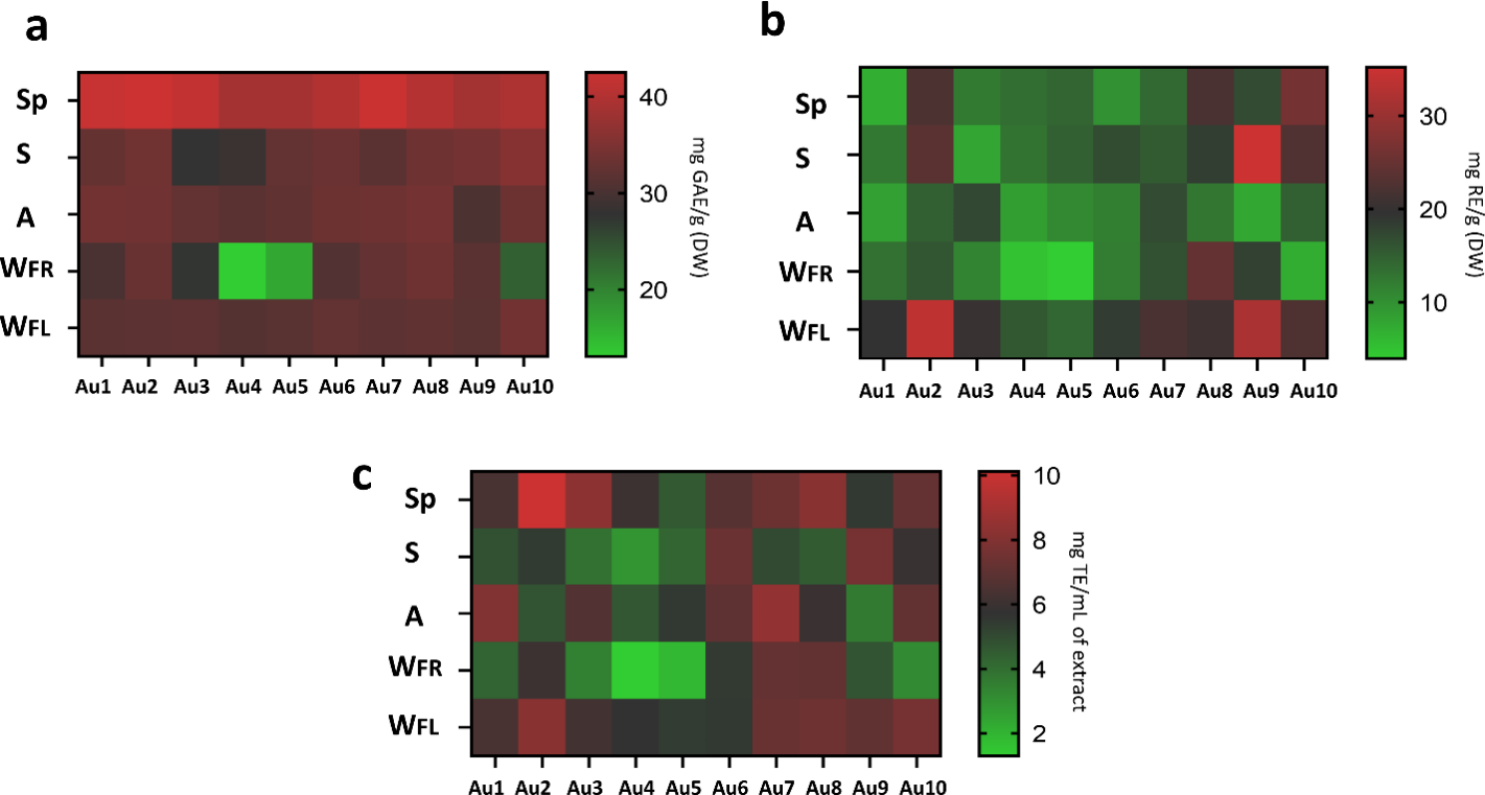
**(a)** Total phenolic content, **(b)** flavonoid content, and **(c)** antioxidant activity of *A. unedo* leaves. Sp = spring, S = summer, A = autumn, W_FR_=winter on fruiting branches, W_FL_=winter flowering branches

In the present study, total polyphenolic and flavonoid content of samples was also evaluated (Fig. 4), and statistical analysis was carried out to compare all data (Fig. S4 and S5) (values and standard deviations are reported in Table S3).

Phenolic content ranged from 8.48 to 42.51 mg of Gallic acid equivalents (GAE)/g (DW) and flavonoid content ranged from 3.96 to 35.16 mg of Rutin equivalents (RE)/g (DW).

Au4 (collected at the highest altitude) was the sample with the lowest content of phenolics in all seasons (Fig. 4A), especially in W_FR_. The difference between Au4 W_FR_ and the other samples was associated to *p* < 0.0001. Except for W_FR_, also Au5 showed a significantly low phenolic content, with values generally comparable to those of Au4. All the samples had the highest phenolic content during Sp (with significant differences associated to *p* < 0.0001), while the lowest content was found always in W_FR_ (Fig. S6).

Regarding total flavonoid content, Au4 and Au5 were clustered also among the samples with the lowest flavonoid content in all seasons (Fig. 4B).

Statistics highlighted that W_FL_ was characterized by the highest flavonoid content in all collection sites. Moreover, W_FR_ had in all cases, except for Au8, significantly lower flavonoid content than W_FL_ (Fig. S7).

**Fig. 5:**
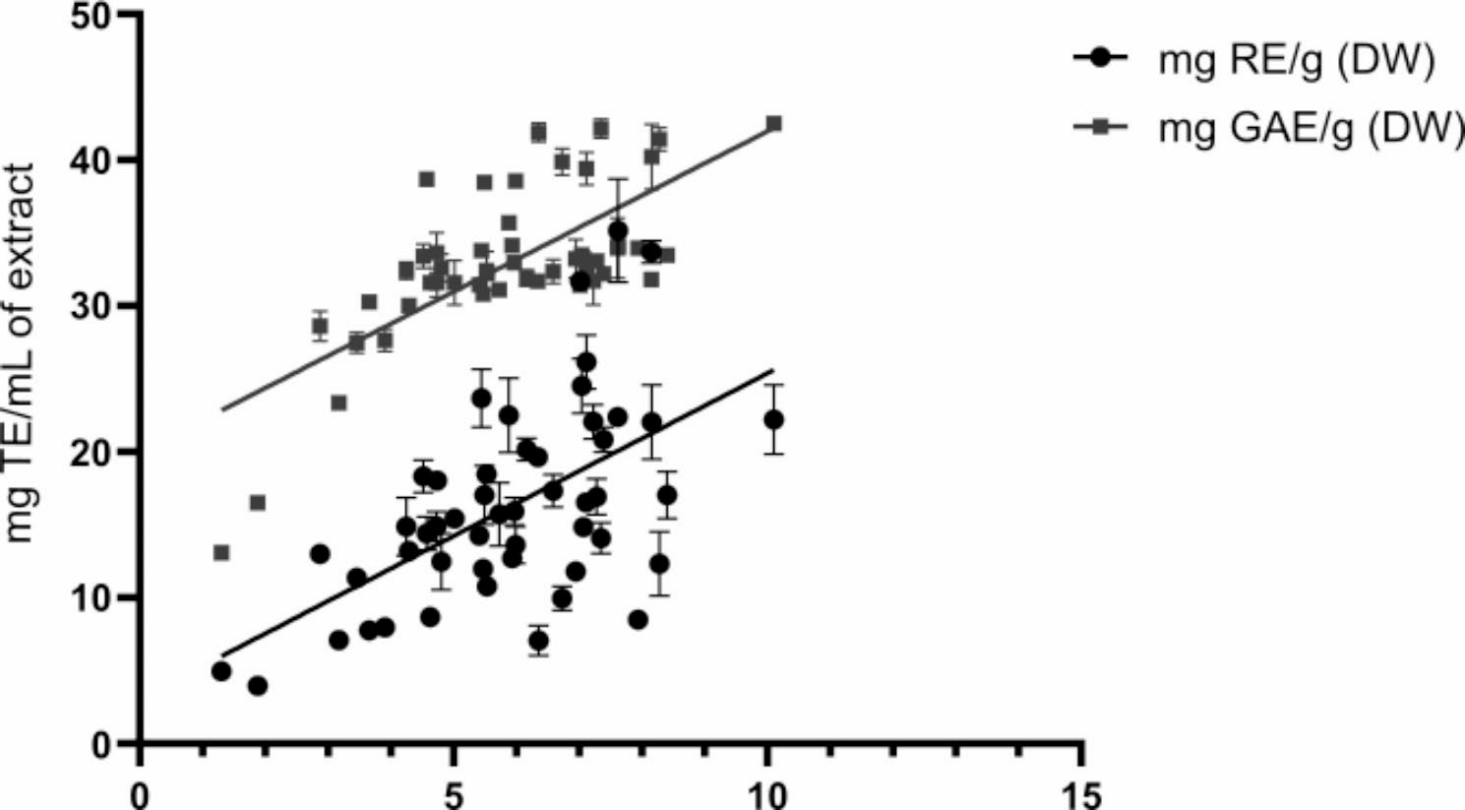
Positive correlation between antioxidant activity (on y axis) and total phenolic and flavonoid content (on x axis). Antioxidant activity was expressed as Trolox equivalents (TE)/mL of extract. Total phenolic content was expressed as mg of gallic acid equivalents (GAE)/g DW. Total flavonoid content was expressed as mg of rutin equivalents (RE)/g DW. Pearson coefficient *r* < 0.0001

Significant differences were also found between the antioxidant activity of the samples, which ranged from 1.3 to 10.1 mg TE/mL of extract (Fig. 4C). According to the statistical analysis, Au4 and Au5 were among the samples with the lowest activity in all seasons (Fig. S8). The lowest content of polyphenols and flavonoids in Au4 and Au5 was reflected in the concomitant lowest antioxidant activity. In support of this observation, total phenolic and flavonoid content were correlated to the antioxidant activity by Pearson test, revealing a direct correlation between the increase in these compounds and antioxidant activity (*r* < 0.0001 in both cases) (Fig. 5), furtherly supporting the importance of these aromatic compounds for the total antioxidant capacity of the extracts.

It was not possible to establish a specific trend in the variation of antioxidant activity across the seasons (Fig. S9). However, in most of the cases, W_FR_ had the lowest antioxidant activity, except for Au7 and Au8, which had antioxidant activity of S lower than W_FR_. The antioxidant activity of W_FL_ was higher and more homogeneous than that observed for W_FR_.

Mean values of temperature (T max and T min), altitude, and precipitation (mm of rain) recorded in all collection sites in the four seasons were correlated in turn to the values of flavonoids, phenolics, and antioxidant activity by Pearson test (Table S4). Total phenolic content showed the highest number of correlations. In particular, in S, A, and W_FR_ the phenolic content significantly correlated to T min, in both cases increasing T min is associated with an increase in phenolics. Phenolic content in A correlated to T max, with a positive trend, and in S and A it decreased at increasing altitude. A negative trend of correlation was established also between phenolics and precipitation in W_FR_ and A.

The only correlation found in the case of flavonoids was between W_FL_ and precipitation and, as in the case of phenolics, the content of flavonoids decreased when precipitation increased. Antioxidant activity in Sp correlated with altitude, while in A and W_FL_ correlated with precipitations, in both cases following a negative trend of correlation. W_FR_ antioxidant activity positively correlated with T min with a positive trend.

### Antibacterial activity

**Fig. 6:**
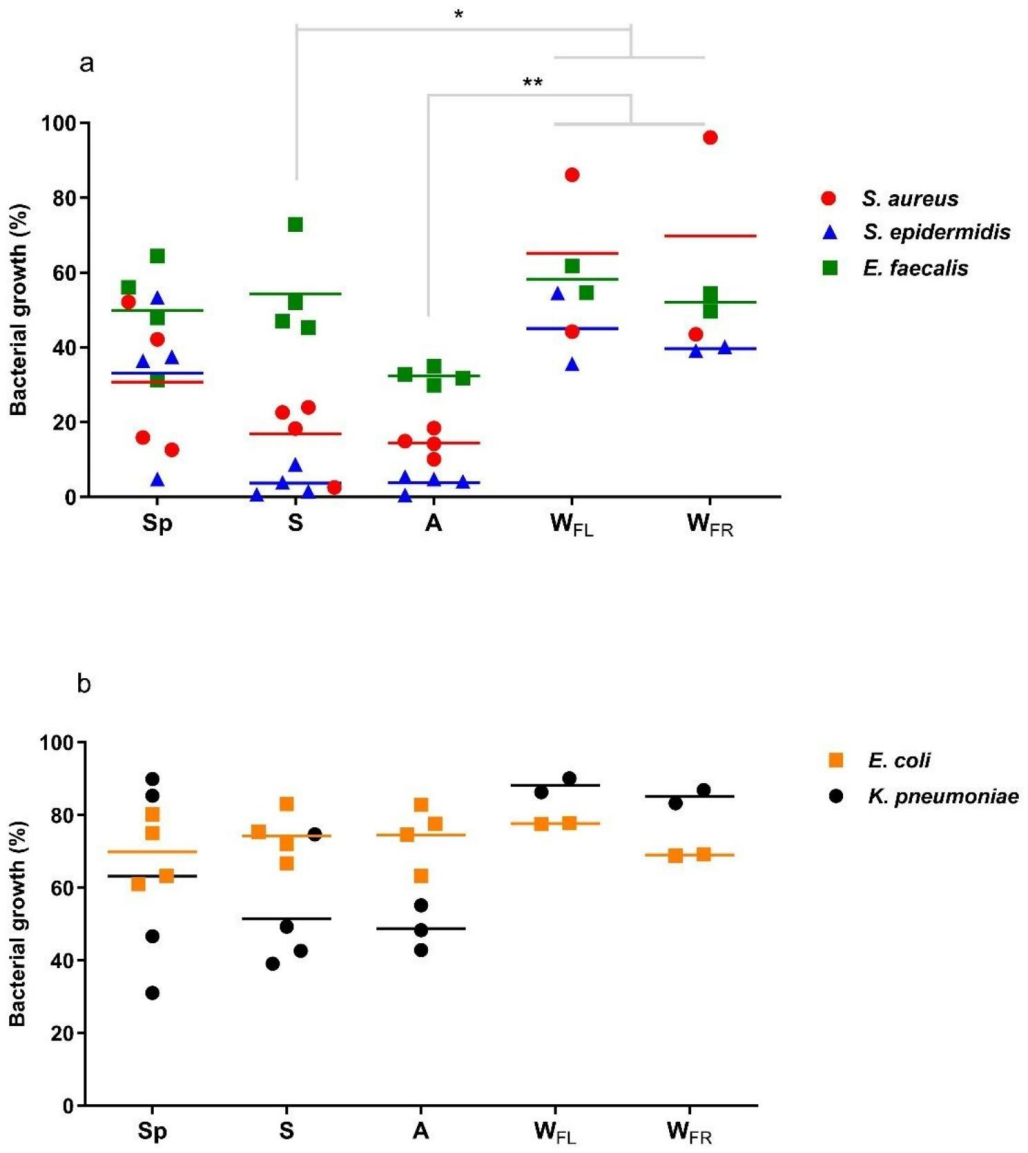
Antibacterial activities of *A. unedo* extracts at 200 µg/mL **(a)** Gram positive bacteria and **(b)** Gram negative bacteria. In the scatter dot plots, symbols are the mean values of each extract obtained in independent experiments, and the lines are the mean values of the grouped categories (Sp, spring; S, summer; A, autumn; W_FL_, winter flowering branches; W_FR_, fruiting branches). Statistically significant differences by ANOVA test (*p* < 0.05) are measured when comparing S and A samples vs. winter flowering/fruiting branches samples in Gram positive strains

For the antibacterial activity four samples for each season were selected based on ^1^ H NMR profile, giving priority to the most phytochemical diverse. In particular, Au1 (Sp); Au2 (W_FR_, Sp, A); Au4 (Sp, S, A); Au5 (W_FR_, Sp, S); Au6 (W_FL_, S); Au8 (S, A); Au9 (W_FL_) and Au10 (A) were assayed in vitro (at a concentration of 200 µg/mL) towards a panel of Gram positive (*S. aureus*, *S. epidermidis*, *E. faecalis*) and Gram negative bacteria (*E. coli*, *K. pneumoniae*). The overall data of bacterial growths are reported in Table S5. Samples collected in A displayed the highest antimicrobial potential regardless of the tested strain, and the difference between these samples and those from W was associated to *p* < 0.05 (Fig. S10A). In addition, when statistical analysis was restricted to *Staphylococcus* species, the most susceptible strains to these plant extracts, data clearly demonstrate the potency of samples collected in A and S (Fig. S10B). Comparing results of the single bacterial species among Gram positive (Fig. 6A) and Gram negative bacteria (Fig. 6B), grouped according to the season of collection, it is worth noting the highest inhibitory activity of samples on the former species. As frequently reported, plant-derived extracts have remarkable antimicrobial potential on Gram positive strains than Gram negative bacteria, related to the inherent lower permeability of the outer membrane and lipopolysaccharides present in their cell wall [15, 16]. These structures increase the permeability threshold of Gram negative strains to many active molecules as well as many classes of clinically effective Gram positive antibiotics [17].

Inhibitory properties of *A. unedo* leaves were finally evaluated by grouping samples according to the site of collection and while there were no differences on the overall anti-bacterial activity, some variations emerged considering only *Staphylococcus* species (Fig. S11). Indeed, Au4 samples displayed the highest inhibitory activity (88.3%) when tested at 200 µg/mL.

Having defined the overall spectrum of the antibacterial activities, the extracts able to inhibit bacterial growths with percentages higher than 50% were further assayed to measure their IC_50_ values. Results are reported in Table 1.

**Table 1:** Anti-staphylococcal activity of the sixteen extracts. Data are expressed as IC_50_ (µg/mL), defined as the concentration giving rise to an inhibition of growth of 50% compared to the positive growth control. Data are reported as mean values and 95% confidence interval

*Label - Season*	*S. aureus* (µg/mL)	*S. epidermidis* (µg/mL)
**Au1 – Sp**	81.9 [67.2–99.9]	72.1 [47.1-110.4]
**Au2 - W**_**FR**_	> 200	151.9 [91.1-253.5]
**Au2 – Sp**	161.8 [137.1–191.0]	80.6 [54.2-119.7]
**Au2 – A**	36.9 [27.9–48.6]	42.3 [36.9–48.4]
**Au4 – Sp**	17.7 [14.6–21.5]	33.6 [30.9–36.6]
**Au4 - S**	34.1 [26.9–43.1]	31.6 [29.2–34.2]
**Au4 – A**	22.6 [18.3–27.8]	24.2 [21.7–26.9]
**Au5 – W**_**FR**_	63.2 [43.3–92.3]	186.5 [140.1-248.3]
**Au5 – Sp**	19.3 [13.6–27.3]	21.4 [19.2–23.8]
**Au5 – S**	21.5 [18.9–24.4]	20.7 [18.2–23.5]
**Au6 – W**_**FL**_	> 200	88.7 [62.7-125.7]
**Au6 – S**	24.1 [20.8–28.0]	18.6 [17.0-20.3]
**Au8 – S**	17.3 [15.0-19.9]	22.0 [17.4–27.8]
**Au8 - A**	20.4 [15.9–26.1]	21.4 [20.1–22.9]
**Au9 – W**_**FL**_	59.2 [50.2–69.7]	123.6 [98.5-155.1]
**Au10 - A**	34.3 [24.9–47.1]	30.5 [27.6–33.7]

Data indicate that samples collected in W (regardless of the W_FL_/W_FR_) were the ones with the lowest antibacterial activity, while those collected in S and A, at the different collection sites, had potent and homogeneous inhibitory profile towards both *Staphylococcal* species, with IC_50_ values ranging from 17.3 to 42.3 µg/mL. These values indicate a significant inhibitory activity of these extracts since the concentration of 100 µg/mL is generally adopted as an endpoint criterion for plant-derived mixtures in all anti‐infective bioassays.

In order to acquire a global overview on the biological properties of the extracts, all samples were also tested for their effects on non-malignant epithelial cells (Table S6). All samples proved to affect cell metabolism at different extent when tested at 200 µg/mL, but at the inhibitory concentrations, none of the extracts was cytotoxic as demonstrated by their CC_50_ values (Table 2). In particular, considering *S. aureus*, the most active extracts within each group (Au4 – Sp, Au8 – S, Au8 – A, Au9 – W_FL_) had CC_50_ values ranging from 112.3 to 355.9 µg/mL. For these samples, the calculated selectivity indexes (SI = CC_50_/IC_50_ ratios) ranged from 5.5 to 9.7, suggesting a preferential inhibitory activity of these samples towards bacterial cells with respect to mammalian cells.

**Table 2:** Selectivity indexes (SI) of the anti-*Staphylococcus* samples

*Label - Season*	*Vero cells* CC_50_* (µg/mL)	*S. aureus* IC_50_ (µg/mL)	SI
**Au4 – Sp**	171.0	17.7	9.7
**Au8 – S**	120.9	17.3	7.0
**Au8 – A**	112.3	20.4	5.5
**Au9 – W**_**FL**_	355.9	59.2	6.0

*Extract concentration required to inhibit Vero cells viability by 50%

## Discussion

In this work, *A. unedo* leaves were studied by ^1^ H NMR metabolomics. The sampling was carried out in Sardinia Island, which is a hotspot for biodiversity, being characterized by a wide range of habitats and a high degree of endemism [18, 19]. Therefore, it represents an extremely diverse and dynamic environment for plants, pushing them to increase and diversify the production of their metabolites in response to a variety of abiotic and biotic stimuli [20].

As emerged from this study, seasons had a remarkable impact on the metabolomic profile of *A. unedo* leaves. S and Sp were characterized by higher concentrations of glucose and sucrose, respectively, which is potentially due to the increased photosynthetic activity occurring during these seasons. Samples of S were also characterized by the highest content of quinic acid, which is considered a precursor of the synthesis of flavonoids and aromatic amino acids [21, 22]. Total phenolic assay evidenced higher concentration of phenolic compounds during Sp, metabolomic analysis suggested that in this season galloyl derivatives might be accumulated.

Catechins and 3-*O*-α-rhamnosyl flavonols increased in W_FL_. Flavonoids exert multiple roles in plants, one of which is to act as UV-filter and radical scavengers, important to counteract the oxidative effects of the high irradiance typical of the hottest seasons [23]. However, the lowering in temperatures, occurring in W, could be another factor potentially responsible for the increased production of flavonols and catechins in this season. As previously reported, the lowering in temperature determines chilling stress in plant tissues, enhancing the production of ROS (reactive oxygen species) and deactivating the ROS quenching systems [24]. Therefore, plants increase the production of antioxidant compounds, especially flavonoids containing catechol structures [25]. Conversely, the lower content of flavonoids found in W_FR_ samples with respect to that of W_FL_ is likely due to the preferential direction of plant lymph and metabolites production towards the ripe fruits rather than the adjacent leaves.

Moreover, according to Castaldi et al. [26], gallocatechin and catechin were found among the most abundant chemicals also in *A. unedo* roots as well as in the extracts of the soil on which the tree was growing. These compounds, released in the soil, are supposed to have a role as denitrification agents through their antimicrobial activity [26].

To the best of our knowledge, this is the first report of 3-*O*-β-methylglucose in *A. unedo*, which resulted generally more concentrated in W. It is considered an osmolyte potentially involved in the freezing-resistance of plants [27], and able to promote flower opening in *Rosa hybrida* by lowering the osmotic potential of plant cell [28]. Interestingly, W was the season characterized by the lowest precipitations so this metabolite may serve also for *A. unedo* as drought resistance osmolyte.

In the majority of the samples, A was characterized by a high content of arbutin. Besides *A. unedo*, arbutin was also found in other species belonging to the same family (i.e. *Vaccinium* spp. or *Arctostaphylos uva-ursi* L.), as well as in species of other families as Rosaceae (i.e. *Pyrus communis* L.), Lamiaceae (i.e. *Origanum majorana* L.), and Myrothamnaceae (i.e. *Myrothamnus flabellifolia* Welw.) [29 ]. The physiological and ecological role of this compound is still under discussion, even though an involvement of arbutin in plant resistance to environmental stress has been proposed. This specialized metabolite is present in plant taxa capable of withstanding extremely low temperatures or extended drought, such as the resurrection plants, which can survive almost completely dehydrated for prolonged periods [30]. The amount of arbutin in *M. flabellifolia* and *A. uva-ursi* is around 25% and 17% DW, respectively [29]. In our work the concentration of arbutin found in *A. unedo* leaves ranged from 0.07 to 5.2% DW, which is coherent with what was found by Jurica et al. [31] who reported a concentration of arbutin of 0.68% DW and 0.27% DW in *A. unedo* leaves collected in two different islands of Croatia.

Arbutin is generally considered one of the most important biomarkers and active principles of *A. unedo* [32], being endowed with anti-inflammatory and antioxidant activities [33], and being a substrate of the enzyme β-glucosidase. It is also widely used in the cosmetic industry as skin depigmenting agent since it counteracts the melanogenesis process by the inhibition of tyrosinase [34]. However, the in vivo bioactivity, especially the diuretic and urinary anti-infective properties, are attributed to a derivative of its hydrolysis, namely hydroquinone [35, 36].

Antioxidant activity was linearly correlated to total phenolic and flavonoid content, although it did not show a clear common trend in relation to seasonality for all samples. However, it was generally observed that Sp and W_FL_ were among the samples characterized by the highest antioxidant activity, while in the other seasons, the variation in antioxidant activity was specific to each sample. In general, it was observed that increasing temperature (both T min and T max) was correlated to an increment in antioxidant compounds (phenolics and flavonoids) and antioxidant activity. Conversely, increasing precipitations and altitude had the opposite effect.

Antibacterial activities resulted primarily affected by the season of harvesting; indeed, samples collected in A displayed the highest inhibitory properties against all tested strains, with remarkable IC_50_ values of 25.7 and 25.4 µg/mL for *S. aureus* and *S. epidermidis*, respectively. Autumn was the season generally yielding the highest level of arbutin and according to Jurica et al. [35] arbutin and its metabolite hydroquinone are active against *E. faecalis* strains.

The results here obtained suggest that A might be considered the balsamic period of *A. unedo* leaves, if the highest concentration of arbutin is sought, in fact, eight out of ten samples had the highest concentration of arbutin in A. Moreover, A samples gave also the highest antibacterial activity against *S. aureus* and *S. epidermidis*. However, phenolic and flavonoids are also important natural active principles, especially known for their antioxidant properties [37, 38]. According to the data here obtained, if the highest content of flavonols and catechins is sought, the leaves should be collected on W_FL_. Conversely, leaves should be harvested in Sp to obtain the highest total phenolics and galloyl derivatives content.

Besides the general influence exerted by seasons on the metabolome of *A. unedo*, some differences have been found from samples harvested in different collection sites, indicating potential involvement of biotic and/or abiotic factors specific to the location. For example, Au4, which was collected at the highest altitude and exposed to the lowest temperatures during the year, had generally less phenolics, flavonoids, and antioxidant activity. Au4 metabolome during W resulted more similar to that of the other samples during Sp. It was observed that flavonols and catechins tend to increase in W, potentially in response to chilling stress, so Au4 might represent a case of acclimatization to the cold. Since it was exposed to low temperatures during all year, it might be less affected by the temperature range shifting from A to W.

Interestingly, Au4 samples were the most active in the antibacterial assay, especially against *Staphylococcus* species in absence of cytotoxic effects on mammalian cells. It is not possible to attribute this activity to the compounds here analyzed, which were present in a lower amount in Au4 than other samples tested.

Except in S, Au3 showed the highest concentration of arbutin, and noteworthy, this sample came from the only one site characterized by limestone soil. It is reported that *A. unedo* prefers siliceous or decarbonated substrates, but grows satisfactorily under a very wide range of soil conditions, with pH varying from 4 to 7, excluding waterlogged soils [39]. Limestone is known to retain moisture in periods of dry weather but allows good drainage during heavy rains. Moreover, calcium and magnesium carbonates, highly characteristic of limestones, increase the global pH of soil being basifying salts.

Furthermore, Au10 showed a low arbutin content in all seasons. Considering that this site is geographically close to Au1, and they were exposed to similar climatic conditions, the particularity of its metabolome is probably ascribable to factors other than the ones considered in this work. It cannot be excluded that Au10 might represent a particular *A. unedo* chemotype or genotype. In fact, although provenience is undoubtedly one of the aspects influencing plant performance and should be considered for plant selection, genotype seems to be the key determining factor [40]. Martins et al. [41] investigated the metabolic responses to drought in strawberry tree genotypes with different geographic origins through a LC-MS untargeted metabolomics proving that sensitivity to water stress was highly genotype-dependent.

## Conclusions

In conclusion, we found that *A. unedo* leaves strongly vary their metabolome according to the harvesting season but also slightly according to the collection site, even if all the samples were collected on the same island (Sardinia). Arbutin is generally more concentrated in autumn, which could be considered the balsamic period of this taxon. Samples collected in this season were also the most active ones as antibacterial and further works are ongoing on *A. unedo* in order to characterize its antibacterial compounds. Moreover, an interesting metabolomic profile enriched in catechins and flavonols was observed in leaves collected in winter on flowering branches which were endowed with high antioxidant potential.

## Materials and methods

### Plant material

**Fig. 7:**
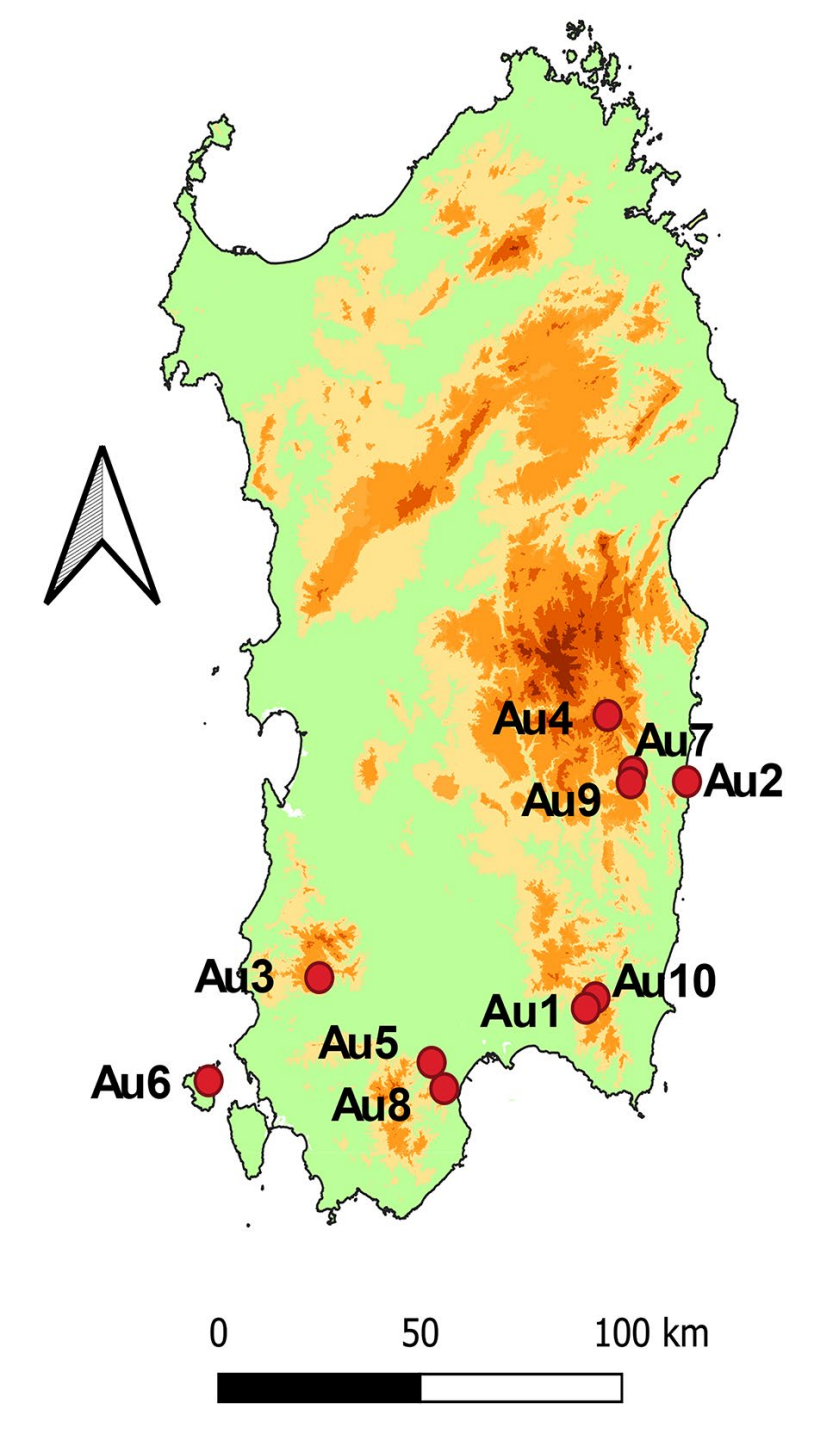
Map of Sardinia reporting the sampling locations. The map was created using QGIS 3.12.0 (QGIS Development Team, 2016) using the DEM file provided by ISPRA and the shape file provided by ISTAT

Leaves of *A. unedo* were harvested in 2018 (metabolomic analysis and biological tests were all performed during 2018–2019), at four time points corresponding to the four seasons, from ten natural populations in the island of Sardinia as reported in Fig. 7. For each location, leaves were collected from ten different trees and then pooled.

Moreover, considering that in winter (W) the plant produces both flowers and fruits (these latter originated from the flowers of the previous year), in this season leaves were harvested from both flowering (W_FL_) and fruiting (W_FR_) branches and they were kept separated from each other.

Species were botanically identified by Cinzia Sanna and voucher specimens (reported in Table 3) were deposited at the General Herbarium of the Department of Life and Environmental Sciences, University of Cagliari (*Herbarium*CAG). After sampling, the fresh plant material was dried in a ventilated stove at 40 °C to constant weight and ground in an electric grinder. Information on collection places (names, altitude, and type of soil), together with vouchers and labels of the samples are reported in Table 3.

**Table 3:** Collection site, altitude (m above sea level), substratum, voucher specimen and label of each sample

Collection site	Altitude	Substratum	Voucher	Label
**Campu Omu**	363	Intrusive rocks of the Paleozoic	CAG 878/V1a	Au1
**Cala Surya**	10	Intrusive rocks of the Paleozoic	CAG 878/V1b	Au2
**Domusnovas**	299	Limestones, dolomites and dolomitic limestones	CAG 878/V1c	Au3
**Gairo Taquisara**	957	Metamorphic rocks	CAG 878/V1d	Au4
**Gutturumannu**	67	Metamorphic rocks	CAG 878/V1e	Au5
**Carloforte**	128	Acid effusive rocks	CAG 878/V1f	Au6
**Porcu e Ludu**	679	Metamorphic rocks	CAG 878/V1g	Au7
**Poggio dei Pini**	121	Intrusive rocks of the Paleozoic	CAG 878/V1h	Au8
**Sant’Antonio**	731	Metamorphic rocks	CAG 878/V1i	Au9
**San Gregorio**	305	Intrusive rocks of the Paleozoic	CAG 878/Vj	Au10

Climatological data considered for statistical analysis are reported in Table S1, and they include: mean of values of minimum and maximum temperatures (T min and T max), and precipitationsexpressed as mm of rain, referred to all seasons. This information was obtained from the database of the climatic monitoring authority of Sardinia available on http://www.sar.sardegna.it/pubblicazioni/riepiloghimensili/mensili.asp.

## Chemical reagents and instruments

Deuterium oxide (D_2_O, 99.90% D) and Deuterated methanol (CD_3_OD, 99.80% D) were purchased from Eurisotop (Cambridge Isotope Laboratories, Inc, France). All the other chemicals were purchased from Sigma-Aldrich Co. (St. Louis, MO, USA). ^1^ H NMR and 2D spectra were recorded on a Varian Inova instrument operating at ^1^ H NMR frequency of 600.13 MHz, for Electrospray Ionization Mass Spectrometry (ESI-MS) analyses WATERS ZQ 4000 (Milford, MA USA) mass spectrometer was used. For fractionation was used Medium Pressure Liquid Chromatography (MPLC) instrument Reveleris® (Büchi, Switzerland) and for biological assays was used microplate reader (Victor™ *X*3 PerkinElmer, Waltham, Massachusetts, United States) and SpeedVac (SPD 101b 230, Savant, Italy).

## Extracts preparation for ^1^ H NMR profiling and biological assays

Samples for ^1^ H NMR profiling were prepared according to Mandrone et al. [42]. For biological assays the samples were prepared following the same procedure performed for ^1^ H NMR profiling, using 1 mL of mixture (1:1) H_2_O and methanol (MeOH). For anti-bacterial and cytotoxicity tests samples were dried in SpeedVac and resuspended in water.

### NMR and ESI-MS experiments and data treatment

For NMR and ESI-MS analyses methods and instruments are as reported in Mandrone et al. [42].

The analysis of ^1^ H NMR profiles of extracts was performed also with the help of an in-house library and comparison with literature [41–44].

Estimation of metabolites amount is reported in Table S2, all the ^1^ H NMR spectra fid together with the metadata were uploaded on Zenodo.

Multivariate analyses were performed using SIMCA software (v. 16.0, Umetrics, Sweden) as reported in Mandrone et al. [42].

### Pre-purification and structural elucidation

Five-hundred g of plant material (obtained by merging material from all samples) were extracted for 72 h with 2 L of MeOH/H_2_O (80:20), filtered on Büchner funnel, and dried in rotary evaporator. The procedure was repeated four times (extract yield 35.16% w/w). Fifty-three g of the dried extract were dissolved in 500 mL of water and successively partitioned with chloroform (CHCl_3_) and ethyl acetate (EtOAc) used in sequence, three times for each step. After anhydrification of the organic fractions, the extracts were dried in rotary evaporator obtaining H_2_O (40 g), EtOAc (5.06 g) and CHCl_3_ (0.25 g) fractions. Since the ^1^ H-NMR profiling revealed that EtOAc fraction contained the metabolites of interest (flavonols and catechins), it was selected for further fractionation using MPLC instrument. The dried EtOAc extract was dissolved in 8 mL of MeOH 60%, injected in C_18_ column (80 g), and eluted with a gradient of H_2_O (solvent A) and MeOH (solvent B). The flow rate was 20 mL/min. The gradient was composed by an isocratic phase of 10 min (10% B), followed by an increase to 20% B in 1 min, an isocratic phase of 20 min (20% B), followed by an increase to 30% B in 1 min, an isocratic phase of 10 min (30% B), followed by an increase to 50% B in 1 min, an isocratic phase of 10 min (50% B), followed by an increase to 70% B in 1 min, an isocratic phase of 5 min (70% B), followed by an increase t 100% B in 1 min kept for the last 5 min. The fractions (Fr) were collected by volume (15 mL each tube), and were successively reduced to eighteen fractions unifying some of them on the basis of the UV-vis chromatogram. Then, the fractions were evaporated using a rotary evaporator and analyzed by ^1^ H NMR. Arbutin was found in fraction 2 (Fr2), catechin together with gallocatechin in Fr4 and Fr5, catechin alone in Fr6. Fr14 contained myricetin 3-*O*-α-rhamnoside, Fr15 quercetin 3-*O*-α-rhamnoside, and Fr17 afzelin. All the ^1^ H-NMR spectra of the following compounds are reported in supplementary Figures S12 and S13.

Afzelin (kaempferol 3-*O*-α-rhamnoside) ^1^H NMR spectral data (600 MHz, CD_3_OD): *δ* 7.75 (2H, *d*, *J* = 8.81 Hz, H-3’, H-5’), 6.92 (2 H, *d*, *J* = 8.81 Hz, H-2’, H-6’), 6.37 (1 H, *d*, *J* = 2.16 Hz, H-8), 6.19 (1 H, *d*, *J* = 2.16 Hz, H-6), 5.36 (1 H, *d*, *J* = 1.74 Hz, H-1’’), 4.20 (1 H, *dd*, *J* = 3.42, 1.74 Hz, H-2’’), 3.68 (1 H, *d*, *J* = 3.42 Hz, H-3’’), 3.31 (1 H, *d*, *J* = 2.69 Hz, H-4’’, H-5’’), 0.90 (3 H, *d*, *J* = 6.29 Hz, H-6’’); ^13^ C NMR spectral data (150 MHz, CD_3_OD): *δ* 159.54 (COH, C-4’), 129.79 (CH, C-3’,C-5’), 120.49 (C, C-1’), 114.38 (CH, C-2’,C-6’), 101.32 (CO, C-1’’), 97.70 (CH, C-6), 92.64 (CH, C-8), 70.91 (CH, C-5’’), 70.03 (CH, C-3’’, C-4’’), 69.76 (CH, C-2’’), 15.56 (CH_3_, C-6’’). Positive ESI-MS *m*/*z*: 353 [M + Na]^+^, 369 [M + K]^+^, calculated as 330.29 for C_14_H_18_O_9_. Negative ESI-MS *m*/*z*: 329 [M – H]^−^.

Arbutin ^1^H NMR spectral data (600 MHz, CD_3_OD): *δ* 6.95 (2H, *d*, *J* = 8.98 Hz, H-3, H-5), 6.68 (2H, *d*, *J* = 8.98 Hz, H-2, H-6), 4.72 (1H, *d*, *J* = 7.7 Hz, H-1’), 3.87 (1 H, *d*, H-2’), 3.68 (1 H, *d*, H-3’), 3.44–3.31 (4 H, *m*, H-4’, H-5’, H-6’a, H-6’b).

Catechin ^1^H NMR spectral data (600 MHz, CD_3_OD): *δ* 6.82 (1H, *d*, *J* = 2.0 Hz, H-2’), 6.74 (1 H, *d*, *J =* 8.1 Hz, H-5’), 6.70 (1 H, *dd*, *J1* = 8.1 Hz, *J2* = 2.0 Hz, H-6’), 5.91 (1 H, *d*, *J* = 2.3 Hz, H-8), 5.83 (1 H, *d*, *J* = 2.3 Hz, H-6), 4.54 (1 H, *d*, *J* = 7.5 Hz, H-2), 3.95 (1 H, *m*, H-3), 2.83 (1 H, *dd*, *J1* = 16.1 Hz, *J2* = 5.4 Hz, H-4), 2.48 (1 H, *dd*, *J1 =* 16.1 Hz, *J2* = 8.2 Hz, H-4).

Gallocatechin ^1^H NMR spectral data (600 MHz, CD_3_OD): *δ* 6.38 (2H, *s*, H -2’, H-6’ ), 5.90 (1 H, *d*, *J* = 2.3 Hz, H-8), 5.84 (1 H, *d*, *J* = 2.3 Hz, H-6), 4.51 (1 H, *d*, *J* = 7.1 Hz, H-2), 3.94 (1 H, *m*, H-3), 2.79 (1 H, *dd*, *J1* = 16.1 Hz, *J2* = 5.3 Hz, H-4), 2.48 (1 H, *dd*, *J1 =* 16.1 Hz, *J2* = 7.8 Hz, H-4).

Myricetin 3-*O*-α-rhamnoside ^1^H NMR spectral data (600 MHz, CD_3_OD): *δ* 6.97 (2H, *s*, H-2’, H-6’), 6.37 (1 H, *d*, *J* = 2.1 Hz, H-8), 6.19 (1 H, *d*, *J* = 2.1 Hz, H-6), 5.34 (1 H, *d*, *J* = 1.7 Hz, H-1’’), 4.21 (1 H, *dd*, *J1* = 3.42 Hz, *J2* = 1.74 Hz, H-2’’), 3.74 (1 H, *dd*, *J1* = 9.44 Hz, *J2* = 3.4 Hz, H-3’’), 3.41 (1 H, *m*, H-4’’), 3.33 (1 H, *m*, H-5’’), 0.93 (3 H, *d*, *J* = 6.2 Hz, H-6’’).

Quercetin 3-*O*-α-rhamnoside ^1^H NMR spectral data (600 MHz, CD_3_OD): *δ* 7.33 (1H, *d*, *J* = 2.1 Hz, H-2’), 7.30 (1 H, *dd*, *J1* = 8.29 Hz, *J2* = 2.1 Hz, H-6’), 6.90 (1 H, *d*, *J* = 8.29 Hz, H-5’), 6.37 (1 H, *d*, *J* = 2.1 Hz, H-8), 6.19 (1 H, *d*, *J* = 2.1 Hz, H-6), 5.30 (1 H, *d*, *J* = 1.7 Hz, H-1’’), 4.21 (1 H, *dd*, *J1* = 3.42, *J2* = 1.74 Hz, H-2’’), 3.74 (1 H, *dd*, *J1* = 9.44 Hz, *J2* = 3.4 Hz), 3.41 (1 H, *m*), 3.33 (1 H, *m*), 0.95 (3 H, *d*, *J* = 6.2 Hz, H-6’’).

### Total phenolic and flavonoid content and DPPH assay

The total phenolic (TPC) (Folin-Ciocalteu method) and total flavonoid content (TFC) of the extracts were assessed by microplate reads Victor™ X3 as described by Chiocchio et al. [45] with slight modifications. For TPC 50 µL of different gallic acid stock solutions were prepared in MeOH (1:1) and mixed with 250 µL of Folin-Ciocalteu reagent (diluted 1:10) and 500 µL of H_2_O. The extracts of Au were diluted in MeOH (1:1) and 50 µL of each stock were mixed with the same reagents as described above, and tested in the assay in duplicate (three independent assays were performed). TFC was determined using rutin to perform the calibration curve following the method described by Mandrone et al. [37]. Thus, TPC was expressed as mg of gallic acid equivalent (GAE) per g of dried plant material (DW), and TFC was expressed as mg of rutin equivalent (RE) per g of dried plant material (DW).

The antioxidant activity of the extracts was determined by DPPH assay as described by Chiocchio et al. [45] with slight modifications. Extracts were diluted in MeOH in order to test different concentrations (ranging from 1.25 to 20 µL/mL in the assay). Methanol was used as negative control, Trolox (Tr) at different concentrations (ranging from 3 to 30 µM) was used as a positive control, and Tr IC_50_ (12.98 µM) was used for the calculation of Trolox equivalents (TE). Total antioxidant activity was expressed as mg of TE per mL of extract.

### Antibacterial activity

The in vitro antibacterial activity of the dried extracts was determined by a standardized microdilution broth assay using a 96-well plate [15], and following the procedures of the Clinical and Laboratory Standard Institute (CLSI). Five reference strains obtained from the American Type Culture Collection were used in the present study: *Staphylococcus aureus* ATCC 25,293, *Staphylococcus epidermidis* (ATCC 12,228), *Enterococcus faecalis* (ATCC 29,212), *Escherichia coli* (ATCC 25,922) and *Klebsiella pneumoniae* (ATCC 9591). For the experiments, a bacterial suspension of 5 × 105 colony forming units per mL (CFU/mL) was prepared in Mueller − Hinton (MH) broth (Sigma-Aldrich, St. Louis, USA) and incubated with the extracts in the range 1.56–200 µg/mL. Following incubation at 37 °C for 24 h, the Abs values were measured at 630 nm; growth percentage values were determined as relative to the positive control (bacterial suspension in regular medium). For extracts demonstrating an inhibitory activity superior to 50% at 200 µg/mL, IC_50_ values were obtained by interpolation on the generated dose-response curves.

### Cell viability assay

Cell viability and proliferation assays were performed on the African green monkey kidney cells (Vero ATCC CCL-81) purchased from American Type culture collection, as previously described [15]. For experiments, cells were seeded into 96-well plates at 104 cells/well, and incubated at 37 °C for 24 h. Then, the extracts were added to the cell monolayer in the range 200 − 1.56 µg/mL. After 48 h of culture, the medium was removed from each well, the monolayer was washed with ice-cold phosphate-buffered saline (PBS), and the cell viability was assessed by a WST8-based assay (CCK-8, Cell Counting Kit-8, Dojindo Molecular Technologies, Rockville, MD, USA). The Abs values were measured at 450 nm and data expressed as the percentage of the cell viability relative to the untreated controls. CC_50_ were obtained by the interpolation of percentage values on the dose-response curves.

### Data analysis

Values were expressed as the mean ± SD of experiments performed in triplicate. Statistical analyses (ANOVA and Person test) were performed as described by Chiocchio et al. 2023 [45] and Chiocchio et al. 2018 [37].

Antibacterial and cytotoxicity assays were performed in triplicate and at least two independent experiments; the IC_50_ and CC_50_ values were determined by interpolation of the dose-response curves generated by plotting the percentages of growth inhibition, relative to the drug-free control (set to 100% of growth), as a function of the tested concentrations (on a logarithm scale).

### Electronic supplementary material

Below is the link to the electronic supplementary material.

Supplementary Material 1

## Data Availability

The datasets generated and/or analyzed during the current study are available in the Zenodo repository [10.5281/zenodo.7817333].

## Abbreviations

A: Autumn
Au: *Arbutus unedo*
COSY: ^1^ H-^1^ H homonuclear Correlation Spectroscopy
DPPH: 2,2-Diphenyl-1picrylhydrazyl
DW: dry weight of plant material
ESI-MS: Electrospray Ionization Mass Spectrometry
Fr: Fractions
GABA: γ-Aminobutyric acid
GAE: Gallic Acid Equivalents
HMBC: Heteronuclear Multiple Bond Correlation
HSQC: Heteronuclear single quantum coherence
J-res: J-resolved Spectroscopy
MeOH: Methanol
MPLC: Medium Pressure Liquid Chromatography
MS: Mass Spectrometry
NMR: Nuclear Magnetic Resonance
OPLS: Orthogonal Partial Least Squares
PBS: Phosphate-buffered saline
PCA: Principal Component Analysis
PLS-DA: Partial Least Squares Discriminant Analysis
S: Summer
Sp: Spring
r: Pearson correlation coefficient
RE: Rutin Equivalents
TE: Trolox Equivalents
TMSP: 3-(trimethylsilyl)-propionic-2,2,3,3-d4 acid sodium salt
W: Winter
W_FL_: Winter flowering branches
W_FR_: Winter fruiting branches
