# Supplementary material for: Metabolomic analysis and bioactivities of Arbutus unedo leaves harvested across the seasons in different natural habitats of Sardinia (Italy)
Source: BMC Plant Biol. 2023 Oct 13;23:490. doi: 10.1186/s12870-023-04497-0 (PMC10571483; doi:10.1186/s12870-023-04497-0)
Supplement: Supplementary file 1 — Supplementary Material 1 [file 12870_2023_4497_MOESM1_ESM.docx]

**Metabolomic analysis and bioactivities of *Arbutus unedo* leaves harvested across the seasons in different natural habitats of Sardinia (Italy)**

Cinzia Sanna^1§^, Ilaria Chiocchio^2§^, Manuela Mandrone^2^*, Francesca Bonvicini^3^, Giovanna A. Gentilomi^3,4^, Simona Trincia^2^, Ferruccio Poli^2^

^1^ Department of Life and Environmental Sciences, University of Cagliari, via Sant‘Ignazio da Laconi 13, 09123 Cagliari, Italy

^2^ Department of Pharmacy and Biotechnology, Alma Mater Studiorum, University of Bologna, Via Irnerio 42, 40126 Bologna, Italy

^3^ Department of Pharmacy and Biotechnology, Alma Mater Studiorum, University of Bologna, Via Massarenti 9, 40138 Bologna, Italy

^4^ Microbiology Unit, IRCCS Azienda Ospedaliero-Universitaria di Bologna, Via Massarenti 9, 40138 Bologna, Italy

^§^ These authors equally contributed to write this work

^*^ Correspondence: Manuela Mandrone

+390512091294 (office)

email: [manuela.mandrone2@unibo.it](mailto:manuela.mandrone2@unibo.it)


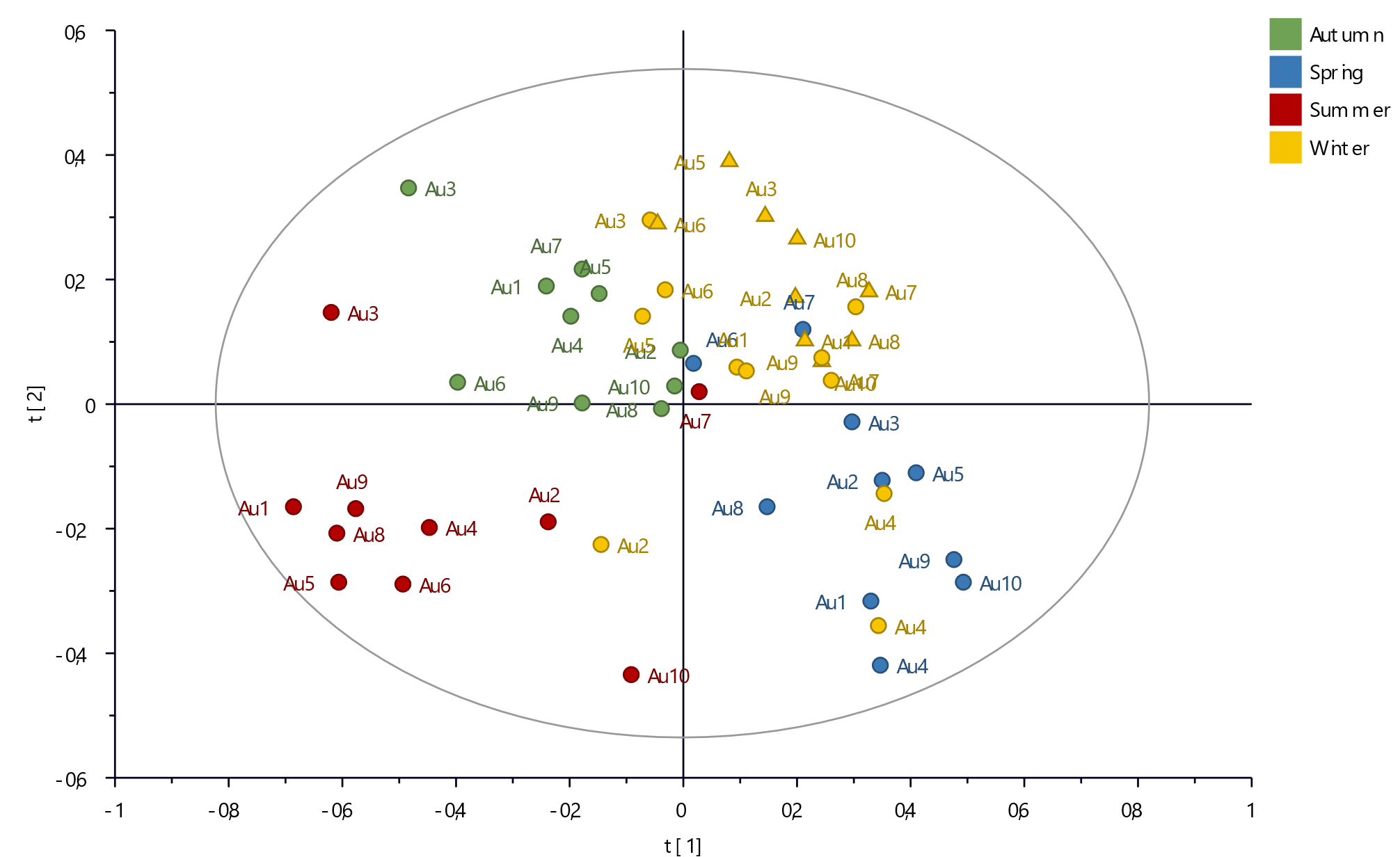


**Figure S1. ^1^H NMR-based PCA Score Scatter Plot** of *A. unedo* leaves harvested in ten different locations (numbers), and in different seasons (indicated by the different colors). Samples from winter fruiting branches (W_FR_) are represented by the yellow triangles. Six components (PCs) maximized the explained 88.6% of the variance in the data set (given by R^2^x(cum)), while the obtained Q^2^(cum) was 67.8%, indicating good predictability (Q^2^ must be equal or higher than 50%).

**
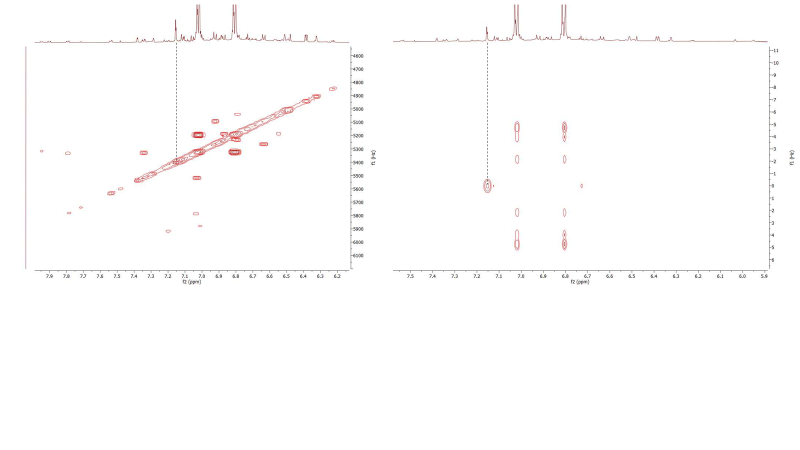
**

**Figure S2. Details of COSY and J-res NMR spectra of *A. unedo* leaves.** On the left side: COSY spectrum of the aromatic region of *A. unedo* leaves, signal at δ 7.15 exhibits no correlations. On the right side: J-res spectrum of the aromatic region of *A. unedo* leaves, signal at δ 7.15 resulted a singlet.


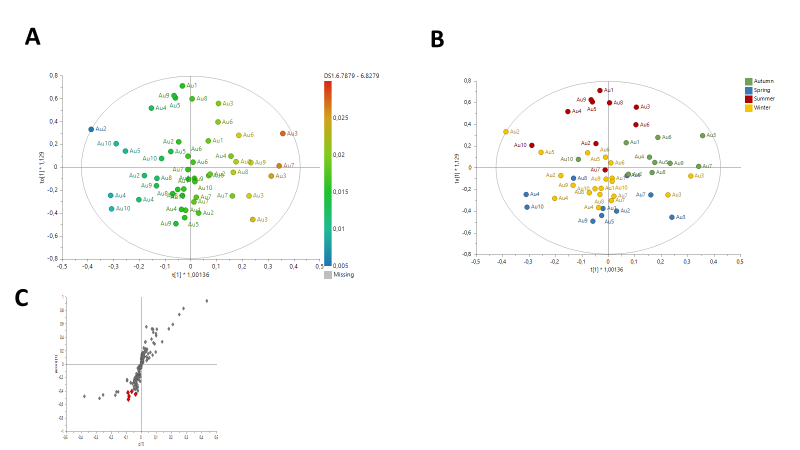


**Figure S3. OPLS model built using as *y* variable the bin at δ 6.79-6.82 (diagnostic signal of arbutin).** **A)** Score scatter plot. Colour gradient is explicative of arbutin content (increasing from blue to red). The samples with the highest arbutin concentration are placed on the positive component t[1]. **B)** Score scatter plot coloured according to season of harvesting. **C)** S-plot indicating the correlation of *x* variables (^1^H NMR signals other than δ 6.79-6.82) to arbutin content (*y* variable). Highlighted in red: signals due to flavonols-α-*O*-rhamnosides, which are the most negatively correlated to arbutin concentration.

**
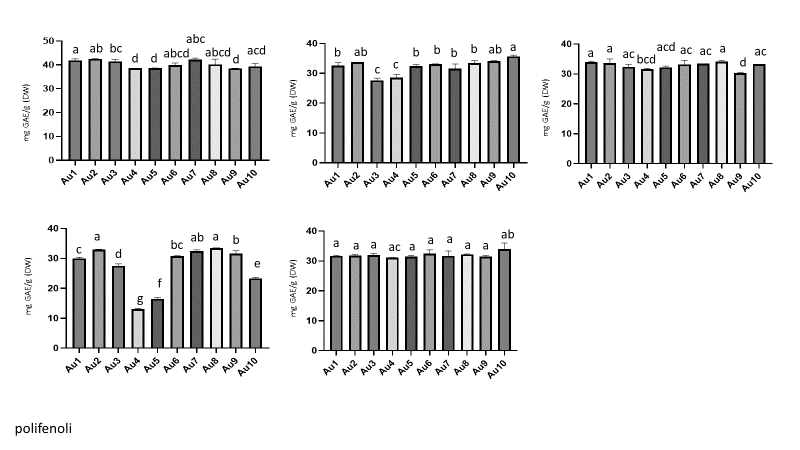
**

**Figure S4. Comparison of total phenolic content (mg GAE/g (DW)) of the samples collected in the different seasons**. Top from left to right: spring, summer, autumn. Bottom from left to right: winter on fruiting branches and winter of flowering branches. On the *x* axis are reported the samples, while different letters within the same graph indicate significant differences in ANOVA test (p < 0.05). Results are expressed as means ± SD of three independent experiments.


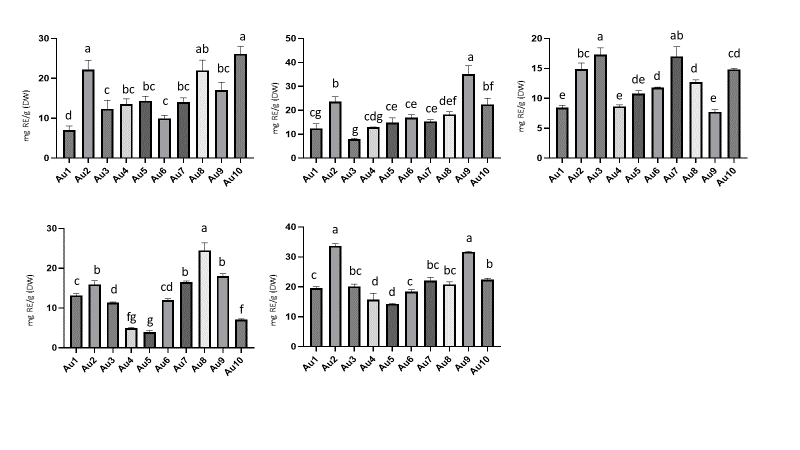


**Figure S5. Comparison of total flavonoid content (mg RE/g (DW)) of the samples collected in the different seasons.** Top from left to right: spring, summer, autumn. Bottom from left to right: winter on fruiting branches and winter of flowering branches. On the *x* axis are indicated the samples, while different letters within the same graph indicate significant differences in ANOVA test (p < 0.05). Results are expressed as means ± SD of three independent experiments.


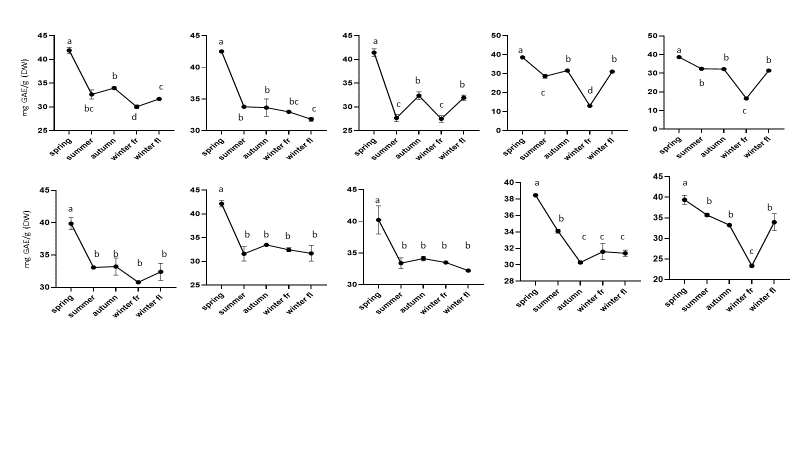


**Figure S6. Variation of total phenolic content (mg GAE/g (DW)) of each sample across the seasons.** Top from left to right: from Au1 to Au5. Bottom from left to right: from Au6 to Au10. On the *x* axis are indicated the seasons (fr= samples on fruiting branches; fl= samples on flowering branches). Different letters within the same graph indicate significant differences in ANOVA test (p < 0.05). Results are expressed as means ± SD of three independent experiments.


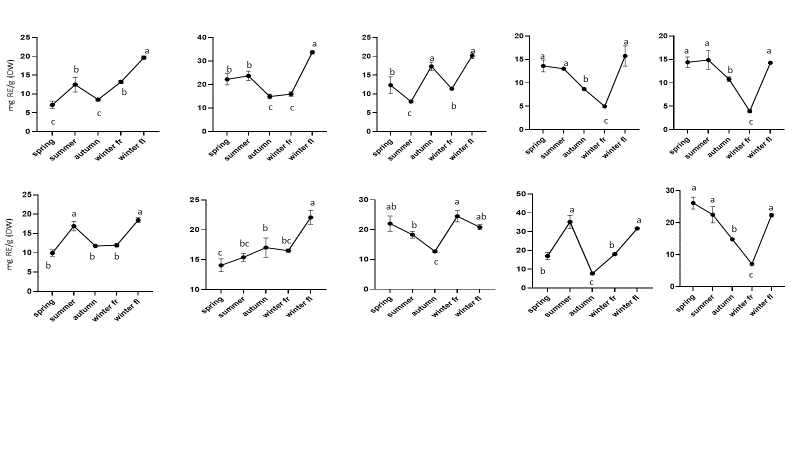


**Figure S7. Variation of total flavonoid content (mg RE/g (DW)) of each sample across the seasons.** Top from left to right: from Au1 to Au5. Bottom from left to right: from Au6 to Au10. On the *x* axis are indicated the seasons (fr= samples on fruiting branches; fl= samples on flowering branches). Different letters within the same graph indicate significant differences in ANOVA test (p < 0.05). Results are expressed as means ± SD of three independent experiments.

**
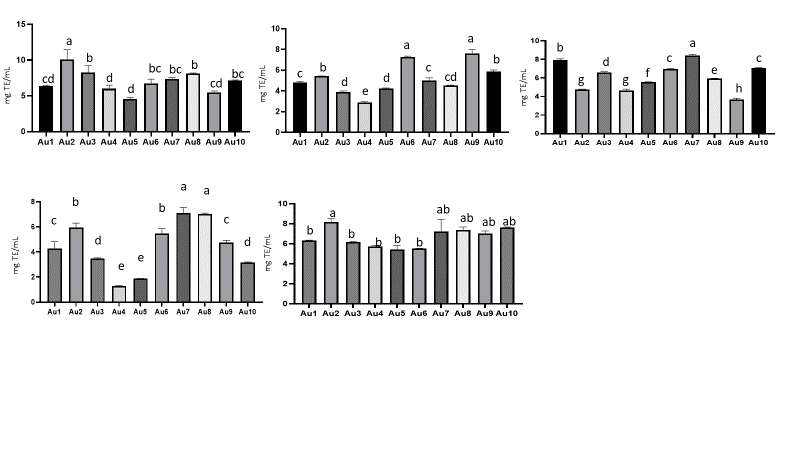
Figure S8. Comparison of DPPH antioxidant activity (mg TE/mL of extract) of the samples collected in the different seasons.** Top from left to right: spring, summer, autumn. Bottom from left to right: winter on fruiting branches and winter of flowering branches. On the *x* axis are indicated the samples, while different letters within the same graph indicate significant differences in ANOVA test (p < 0.05). Results are expressed as means ± SD of three independent experiments.


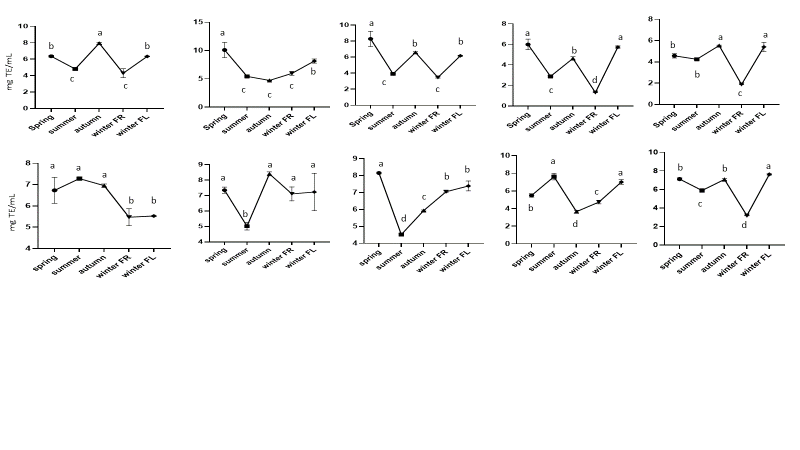


**Figure S9. Variation of DPPH antioxidant activity (mg TE/g of extract) of each sample across the seasons.** Top from left to right: from Au1 to Au5. Bottom from left to right: from Au6 to Au10. On the *x* axis are indicated the seasons (FR= samples on fruiting branches; FL= samples on flowering branches). Different letters within the same graph indicate significant differences in ANOVA test (p < 0.05). Results are expressed as means ± SD of three independent experiments.

**Figure S10.** Activity of the samples per season. Bacterial growths of all tested bacteria regardless the strains (A) and of *Staphylococcus* species (B) when incubated with 200 µg/mL of extract. Data are expressed as percentage values (means ± SD) relative to the positive growth controls (bacteria grown in regular medium). Statistically significant differences by ANOVA test (*p* < 0.05) are measured between autumn/summer samples and those obtained in winter.

**Figure S11.** Activity of the samples per location of harvesting. Bacterial growths of all tested bacteria regardless the strains (A) and of *Staphylococcus* species (B) when incubated with 200 µg/mL of extract. Data are expressed as percentage values (means ± SD) relative to the positive growth controls (bacteria grown in regular medium).

**
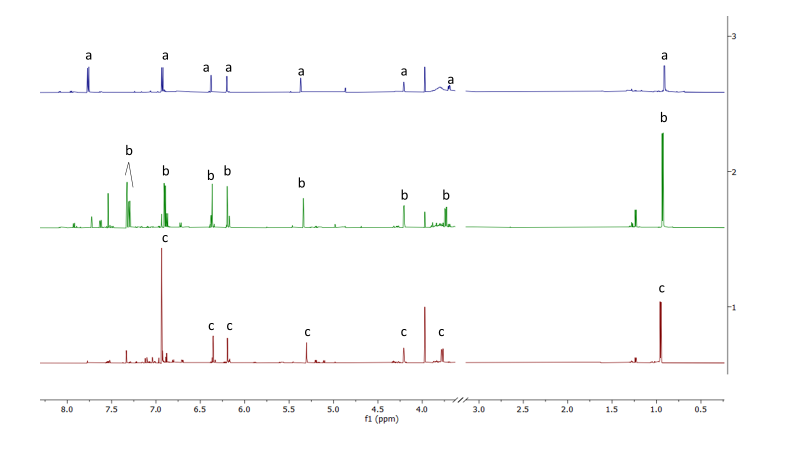
Figure S12.** ^1^H-NMR spectra of partially purified compounds **a)** afzelin **b)** quercetin 3-*O*-α-rhamnoside  **c)** myricetin 3-*O*-α-rhamnoside

**
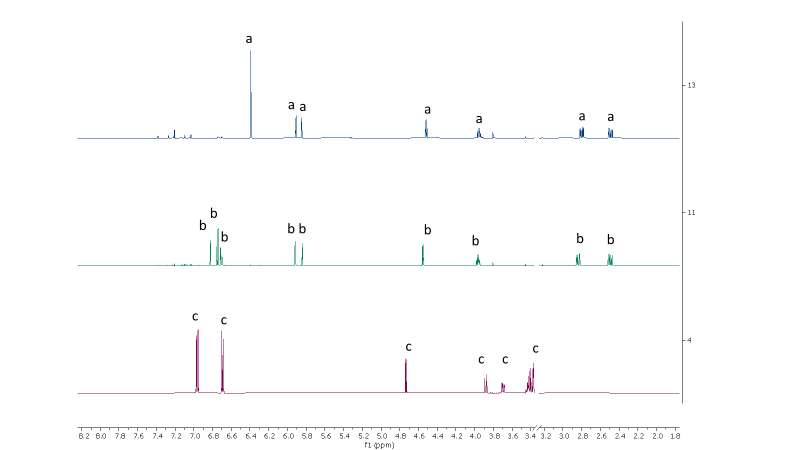
**

**Figure S13.** ^1^H-NMR spectra of partially purified compounds **a)** gallocatechin **b)** catechin **c)** arbutin.

|  | | ***Spring*** | | ***Summer*** | |  | ***Autumn*** | | ***Winter*** | |
| --- | --- | --- | --- | --- | --- | --- | --- | --- | --- | --- |
| **Collection site** | **Label** | **T °C**  **(min-max)** | **Rain**  **(mm)** | **T °C**  **(min-max)** | **Rain**  **(mm)** |  | **T °C**  **(min-max)** | **Rain**  **(mm)** | **T °C**  **(min-max)** | **Rain**  **(mm)** |
| Campu Omu | Au1 | 8-18 | 36-40 | 14-24 | 61-80 |  | 12-20 | 321-360 | 8-12 | 2-10 |
| Cala Surya | Au2 | 10-16 | 46-50 | 18-26 | 21-40 |  | 16-24 | 501-600 | 8-16 | 2-10 |
| Domusnovas | Au3 | 10-18 | 46-50 | 14-24 | 61-80 |  | 14-20 | 141-160 | 6-14 | 31-40 |
| Gairo Taquisara | Au4 | 4-14 | 51-55 | 12-22 | 161-179 |  | 8-16 | 141-161 | 2-9 | 41-50 |
| Gutturumannu | Au5 | 10-18 | 36-40 | 14-26 | 41-60 |  | 14-20 | 501-600 | 8-14 | 2-10 |
| Carloforte | Au6 | 12-22 | 36-40 | 18-28 | 21-40 |  | 16-24 | 181-200 | 8-14 | 21-30 |
| Porcu e ludu | Au7 | 6-12 | 61-65 | 12-22 | 141-160 |  | 10-18 | 201-240 | 6-10 | 11-20 |
| Poggio dei Pini | Au8 | 12-18 | 31-35 | 18-26 | 21-40 |  | 16-24 | 501-601 | 8-14 | 2-10 |
| Sant’Antonio | Au9 | 8-12 | 61-65 | 12-22 | 141-160 |  | 10-18 | 201-240 | 6-10 | 11-20 |
| San Gregorio | Au10 | 8-18 | 31-35 | 16-24 | 41-60 |  | 12-20 | 321-360 | 8-12 | 2-10 |

**Table S1.** Mean of values of Temperature (min and max) and precipitation (mm of rain) recorded in all collection sites in the four seasons.

| arbutin  (δ 6.81) | spring | summer | autumn | winter FR | winter FL |
| --- | --- | --- | --- | --- | --- |
| Au1 | 32.1 | 29.0 | 29.6 | 24.0 | 33.1 |
| Au2 | 37.4 | 31.1 | 31.4 | 24.3 | 10.9 |
| Au3 | 52.0 | 25.6 | 51.0 | 32.4 | 43.8 |
| Au4 | 12.8 | 17.1 | 24.4 | 6.1 | 4.8 |
| Au5 | 28.1 | 26.7 | 30.3 | 4.8 | 21.7 |
| Au6 | 31.8 | 44.2 | 47.3 | 22.7 | 29.2 |
| Au7 | 8.6 | 25.4 | 35.0 | 30.5 | 32.0 |
| Au8 | 22.8 | 27.7 | 33.4 | 30.5 | 27.4 |
| Au9 | 22.9 | 35.1 | 24.3 | 26.4 | 34.6 |
| Au10 | 15.2 | 16.7 | 19.2 | 17.5 | 31.8 |
| sucrose  (δ 5.4) | spring | summer | autumn | winter FR | winter FL |
| Au1 | 99.2 | 25.6 | 33.4 | 54.9 | 72.4 |
| Au2 | 104.2 | 66.3 | 62.5 | 61.9 | 74.4 |
| Au3 | 98.9 | 11.6 | 17.3 | 40.6 | 34.1 |
| Au4 | 101.3 | 28.9 | 33.4 | 32.0 | 10.7 |
| Au5 | 81.9 | 45.6 | 34.7 | 10.7 | 35.8 |
| Au6 | 57.5 | 65.2 | 60.0 | 35.2 | 46.4 |
| Au7 | 18.3 | 47.2 | 28.7 | 52.2 | 71.1 |
| Au8 | 73.7 | 28.9 | 49.0 | 52.2 | 72.1 |
| Au9 | 95.0 | 34.6 | 31.1 | 61.7 | 75.0 |
| Au10 | 94.0 | 78.9 | 46.2 | 51.0 | 75.4 |
| glucose  (δ 5.2; 4.6) | spring | summer | autumn | winter FR | winter FL |
| Au1 | 11.7 | 19.7 | 10.7 | 6.0 | 7.9 |
| Au2 | 8.5 | 9.2 | 5.1 | 10.4 | 7.5 |
| Au3 | 11.2 | 12.1 | 10.7 | 7.0 | 12.0 |
| Au4 | 10.7 | 13.8 | 5.0 | 3.8 | 10.3 |
| Au5 | 7.4 | 14.8 | 5.8 | 3.1 | 13.4 |
| Au6 | 9.8 | 17.4 | 9.3 | 8.0 | 9.5 |
| Au7 | 2.3 | 10.2 | 6.8 | 6.5 | 8.6 |
| Au8 | 13.8 | 19.8 | 7.06 | 5.8 | 9.8 |
| Au9 | 5.8 | 21.9 | 8.6 | 7.8 | 13.6 |
| Au10 | 8.3 | 8.1 | 7.9 | 3.2 | 11.4 |
| methyl-glucose (δ 4.3) | spring | summer | autumn | Winter FR | Winter FL |
| Au1 | 17.0 | 14.2 | 20.9 | 24.5 | 28.6 |
| Au2 | 22.6 | 17.1 | 22.8 | 29.5 | 22.1 |
| Au3 | 17.4 | 8.0 | 14.0 | 16.0 | 18.5 |
| Au4 | 17.3 | 13.5 | 13.5 | 8.7 | 27.13 |
| Au5 | 18.2 | 8.8 | 15.8 | 5.5 | 19.1 |
| Au6 | 18.7 | 11.6 | 27.1 | 19.1 | 23.9 |
| Au7 | 2.3 | 19.1 | 16.6 | 24.0 | 27.6 |
| Au8 | 16.6 | 8.0 | 16.0 | 22.3 | 21.6 |
| Au9 | 16.3 | 15.3 | 10.5 | 18.6 | 22.7 |
| Au10 | 20.6 | 18.2 | 22.4 | 7.4 | 25.2 |
| quercetin-3-*O*-rhamnoside (δ 0.91) | spring | summer | autumn | winter FR | winter FL |
| Au1 | 4.4 | 5.7 | 4.1 | 5.1 | 10.0 |
| Au2 | 11.8 | 12.4 | 8.1 | 14.2 | 12.5 |
| Au3 | 7.7 | 3.7 | 7.9 | 5.9 | 9.5 |
| Au4 | 6.8 | 5.1 | 4.1 | 1.5 | 2.3 |
| Au5 | 8.5 | 8.0 | 6.1 | 2.3 | 6.6 |
| Au6 | 5.1 | 7.0 | 4.8 | 5.8 | 8.4 |
| Au7 | 1.6 | 6.2 | 0.7 | 7.9 | 9.7 |
| Au8 | 9.6 | 7.9 | 6.3 | 7.9 | 10.7 |
| Au9 | 7.3 | 13.5 | 3.0 | 9.2 | 12.3 |
| Au10 | 15.5 | 11.3 | 7.8 | 7.1 | 11.1 |
| gallocatechin (δ 6.48) | spring | summer | autumn | winter FR | winter FL |
| Au1 | 2.5 | 1.3 | 3.8 | 2.9 | 5.3 |
| Au2 | 5.1 | 5.5 | 3.8 | 4.9 | 5.0 |
| Au3 | 2.6 | n.d. | 1.3 | 1.2 | 4.1 |
| Au4 | 2.4 | n.d | 2.1 | 0.7 | 0.6 |
| Au5 | 1.7 | 0.9 | 2.1 | 0.6 | 2.3 |
| Au6 | 1.7 | 2.1 | 4.5 | 2.0 | 3.0 |
| Au7 | 0.6 | 3.1 | 3.6 | 3.8 | 4.6 |
| Au8 | 2.0 | n.d. | 4.2 | 3.8 | 5.7 |
| Au9 | 2.0 | 1.7 | 2.4 | 5.0 | 4.5 |
| Au10 | 1.3 | 1.2 | 3.1 | 2.5 | 4.7 |
| afzalin (δ 7.8) | spring | summer | autumn | winter FR | winter FL |
| Au1 | 0.8 | 0.9 | 1.0 | n.d. | 1.2 |
| Au2 | 0.7 | 0.7 | 0.6 | 1.0 | 0.7 |
| Au3 | 1.2 | 0.6 | 0.8 | 0.2 | 0.7 |
| Au4 | 0.3 | 0.4 | 0.5 | n.d. | 0.1 |
| Au5 | 0.5 | 0.5 | 0.4 | 0.1 | 0.6 |
| Au6 | 0.9 | 1.4 | n.d. | 0.4 | 0.7 |
| Au7 | 0.7 | 0.6 | 0.5 | 0.5 | 0.6 |
| Au8 | 0.9 | 0.6 | 0.6 | 0.5 | 0.6 |
| Au9 | 0.4 | 0.7 | 0.2 | 0.7 | 0.8 |
| Au10 | 0.8 | 0.9 | 0.7 | 0.4 | 0.8 |
| myricetin-3-*O*-rhamnoside (δ 0.93) | spring | summer | autumn | winter FR | winter FL |
| Au1 | 2.9 | 2.9 | 3.3 | 4.4 | 3.8 |
| Au2 | 4.8 | 3.9 | 3.0 | 6.8 | 7.4 |
| Au3 | 3.3 | 2.7 | 5.2 | 3.5 | 5.3 |
| Au4 | 4.0 | 3.1 | 2.8 | 1.3 | 1.5 |
| Au5 | 5.2 | 3.1 | 3.4 | 1.5 | 3.6 |
| Au6 | 3.9 | 3.4 | 3.1 | 3.6 | 4.7 |
| Au7 | 1.2 | 4.6 | 4.8 | 4.5 | 5.9 |
| Au8 | 5.7 | 5.4 | 3.8 | 4.5 | 6.7 |
| Au9 | 5.4 | 9.6 | 2.4 | 5.6 | 7.3 |
| Au10 | 10.4 | 7.8 | 5.9 | 4.9 | 4.8 |
| catechin  (δ 6.89; 6.85) | spring | summer | autumn | winter FR | winter FL |
| Au1 | 6.2 | 3.5 | 8.6 | 9.6 | 4.1 |
| Au2 | 8.6 | 5.5 | 5.9 | 7.7 | 8.1 |
| Au3 | 5.7 | 1.9 | 3.6 | 7.9 | 3.5 |
| Au4 | 5.0 | 3.2 | 3.8 | 1.7 | 0.2 |
| Au5 | 5.4 | 4.0 | 5.1 | 6.4 | 2.1 |
| Au6 | 4.1 | 4.6 | 6.8 | 8.2 | 5.6 |
| Au7 | 1.2 | 5.2 | 7.2 | 10.7 | 6.8 |
| Au8 | 5.2 | 4.6 | 7.2 | 9.8 | 6.8 |
| Au9 | 5.6 | 5.0 | 3.8 | 8.4 | 9.3 |
| Au10 | 4.5 | 4.0 | 5.5 | 12.5 | 6.4 |

**Table S2.** Results of semi-quantitative analysis performed by ^1^H NMR, values are expressed in mg/g of DW. The chemical shift of the diagnostic signal used for quantification is indicated in brackets; n.d.= not detected.

| Sample and Season | DPPH | Total flavonoids | Total phenolics |
| --- | --- | --- | --- |
| SPRING | | | |
| Au1 | 6.36 ± 0.11 | 7.07 ± 1.02 | 41.89 ± 0.65 |
| Au2 | 10.11 ± 1.34 | 22.20 ± 2.36 | 42.51 ± 0.12 |
| Au3 | 8.28 ± 0.95 | 12.33 ± 2.20 | 41.43 ± 0.79 |
| Au4 | 5.99 ± 0.52 | 13.60 ± 1.28 | 38.57 ± 0.01 |
| Au5 | 4.58 ± 0.20 | 14.40 ± 1.13 | 38.66 ± 0.01 |
| Au6 | 6.73 ± 0.61 | 9.96 ± 0.83 | 39.85 ± 0.93 |
| Au7 | 7.35 ± 0.20 | 14.07 ± 1.07 | 42.15 ± 0.65 |
| Au8 | 8.16 ± 0.05 | 22.03 ± 2.54 | 40.22 ± 2.23 |
| Au9 | 5.49 ± 0.19 | 17.03 ± 2.02 | 38.47 ± 0.08 |
| Au10 | 7.12 ± 0.12 | 26.17 ± 1.85 | 39.39 ± 1.11 |
| SUMMER | | | |
| Au1 | 4.81 ± 0.12 | 12.46 ± 1.94 | 32.63 ± 0.96 |
| Au2 | 5.45 ± 0.01 | 23.68 ± 1.99 | 33.77 ± 0.01 |
| Au3 | 3.91 ± 0.08 | 7.98 ± 0.36 | 27.66 ± 0.77 |
| Au4 | 2.88 ± 0.09 | 13.00 ± 0.05 | 28.60 ± 1.04 |
| Au5 | 4.25 ± 0.04 | 14.87 ± 1.98 | 32.40 ± 0.58 |
| Au6 | 7.28 ± 0.08 | 16.93 ± 1.24 | 33.08 ± 0.11 |
| Au7 | 5.02 ± 0.25 | 15.41 ± 0.65 | 31.59 ± 1.51 |
| Au8 | 4.52 ± 0.05 | 18.32 ± 1.12 | 33.40 ± 0.84 |
| Au9 | 7.62 ± 0.37 | 35.16 ± 3.53 | 34.10 ± 0.23 |
| Au10 | 5.88 ± 0.15 | 22.50 ± 2.54 | 35.69 ± 0.42 |
| AUTUMN | | | |
| Au1 | 7.94 ± 0.12 | 33.96 ± 0.24 | 8.48 ± 0.38 |
| Au2 | 4.73 ± 0.06 | 14.90 ± 1.00 | 33.63 ± 1.37 |
| Au3 | 6.59 ± 0.09 | 17.32 ± 1.12 | 32.35 ± 0.81 |
| Au4 | 4.63 ± 0.17 | 8.67 ± 0.25 | 31.61 ± 0.20 |
| Au5 | 5.53 ± 0.04 | 10.77 ± 0.51 | 32.21 ± 0.41 |
| Au6 | 6.95 ± 0.08 | 11.78 ± 0.10 | 33.22 ± 1.32 |
| Au7 | 8.41 ± 0.13 | 17.03 ± 1.61 | 33.48 ± 0.01 |
| Au8 | 5.93 ± 0.04 | 12.71 ± 0.39 | 34.12 ± 0.37 |
| Au9 | 3.66 ± 0.16 | 7.75 ± 0.35 | 30.29 ± 0.15 |
| Au10 | 7.07 ± 0.09 | 14.85 ± 0.14 | 33.24 ± 0.01 |
| WINTER FR | | | |
| Au1 | 4.29 ± 0.54 | 13.19 ± 0.44 | 30.02 ± 0.37 |
| Au2 | 5.97 ± 0.34 | 15.93 ± 0.94 | 32.97 ± 0.09 |
| Au3 | 3.46 ± 0.07 | 11.37 ± 0.18 | 27.47 ± 0.70 |
| Au4 | 1.31 ± 0.01 | 4.97 ± 0.16 | 13.08 ± 0.31 |
| Au5 | 1.88 ± 0.01 | 3.96 ± 0.37 | 16.52 ± 0.43 |
| Au6 | 5.47 ± 0.40 | 11.97 ± 0.44 | 30.80 ± 0.11 |
| Au7 | 7.11 ± 0.45 | 16.52 ± 0.28 | 32.45 ± 0.43 |
| Au8 | 7.05 ± 0.06 | 24.50 ± 1.89 | 33.49 ± 0.05 |
| Au9 | 4.74 ± 0.20 | 18.04 ± 0.59 | 31.60 ± 0.99 |
| Au10 | 7.17 ± 0.33 | 7.10 ± 0.18 | 23.34 ± 0.27 |
| WINTER FL | | | |
| Au1 | 6.34 ± 0.03 | 19.63 ± 0.44 | 31.68 ± 0.19 |
| Au2 | 8.15 ± 0.37 | 33.71 ± 0.76 | 31.79 ± 0.31 |
| Au3 | 6.17 ± 0.06 | 20.16 ± 0.76 | 31.93 ± 0.57 |
| Au4 | 5.73 ± 0.12 | 15.72 ± 2.16 | 31.08 ± 0.05 |
| Au5 | 5.41 ± 0.43 | 14.27 ± 0.18 | 31.44 ± 0.45 |
| Au6 | 5.53 ± 0.02 | 18.46 ± 0.63 | 32.41 ± 1.32 |
| Au7 | 7.23 ± 1.21 | 22.07 ± 1.16 | 31.70 ± 1.64 |
| Au8 | 7.39 ± 0.29 | 20.84 ± 0.84 | 32.22 ± 0.12 |
| Au9 | 7.02 ± 0.27 | 31.67 ± 0.20 | 31.41 ± 0.38 |
| Au10 | 7.62 ± 0.07 | 22.37 ± 0.51 | 33.96 ± 2.05 |

**Table S3.** Mean and standard deviation of antioxidant activity (DPPH) (mg TE/mL of extract), total flavonoids (mg RE/g (DW)) and total phenolics (mg GAE/g (DW)).

| PHENOLIC CONTENT | | | | | |
| --- | --- | --- | --- | --- | --- |
|  | **Spring** | **Summer** | **Autumn** | **Winter FR** | **Winter FL** |
| Altitude | NS | r < 0.0497 | r < 0.042 | NS | NS |
| Tmin | NS | r < 0.0155 | r < 0.0070 | r < 0.0037 | NS |
| Tmax | NS | NS | r < 0.0014 | NS | NS |
| Rain | NS | NS | r < 0.0467 | r < 0.0428 | NS |
| FLAVONOID CONTENT | | | | | |
| Altitude | NS | NS | NS | NS | NS |
| Tmin | NS | NS | NS | NS | NS |
| Tmax | NS | NS | NS | NS | NS |
| Rain | NS | NS | NS | NS | r < 0.0083 |
| DPPH | | | | | |
| Altitude | r < 0.0487 | NS | NS | NS | NS |
| Tmin | NS | NS | NS | r < 0.0202 | NS |
| Tmax | NS | NS | NS | NS | NS |
| Rain | NS | NS | r < 0.0035 | NS | r < 0.0005 |

**Table S4.** Results of Pearson correlation test between total phenolic content, flavonoid content, antioxidant activity and the environmental parameters of the locations. If Pearson coefficient r was significant, the value is reported, NS = non-significant.

| *Label - Season* | *S. aureus* | *S. epidermidis* | *E. faecalis* | *E. coli* | *K. pneumoniae* |
| --- | --- | --- | --- | --- | --- |
| Au1 – Sp | 66.5±1.0 | 48.5±1.7 | 64.4±6.4 | 80.2±3.2 | 89.9±1.8 |
| Au2 - W_FR_ | 56.2±7.0 | 49.1±1.0 | 54.4±1.0 | 69.2±1.7 | 86.9±1.9 |
| Au2 – Sp | 58.3±4.3 | 48.7±4.3 | 47.9±4.6 | 75.0±2.4 | 85.3±1.1 |
| Au2 – A | 15.6±8.1 | 4.8±0.5 | 34.9±0.1 | 74.6±1.5 | 42.8±4.0 |
| Au4 – Sp | 16.5±5.1 | 0.1±4.5 | 56.0±0.9 | 63.3±1.4 | 31.0±2.5 |
| Au4 - S | 23.9±7.9 | 8.7±7.5 | 72.9±2.5 | 93.1±1.4 | 74.7±0.6 |
| Au4 – A | 17.3±4.2 | 4.2±6.4 | 32.8±1.0 | 63.3±0.9 | 48.3±0.9 |
| Au5 – W_FR_ | 55.6±5.1 | 49.6±3.6 | 49.6±3.5 | 68.8±1.6 | 83.3±1.7 |
| Au5 – Sp | 24.0±8.5 | 5.7±3.4 | 31.3±2.5 | 61.0±0.9 | 46.7±0.1 |
| Au5 – S | 18.3±2.0 | 1.5±1.4 | 45.2±5.1 | 66.7±1.6 | 42.6±2.5 |
| Au6 – W_FL_ | 57.2±5.1 | 47.5±3.8 | 61.7±3.3 | 77.8±5.9 | 90.1±1.5 |
| Au6 – S | 2.5±1.9 | 1.5±1.3 | 52.0±1.8 | 72.0±1.9 | 39.1±1.5 |
| Au8 – S | 25.4±1.1 | 4.7±6.6 | 47.0±10.6 | 75.4±0.9 | 49.3±1.7 |
| Au8 - A | 18.4±9.9 | 2.3±1.4 | 31.8±1.3 | 77.6±2.0 | 55.2±3.2 |
| Au9 – W_FL_ | 66.9±4.2 | 59.2±9.2 | 54.6±9.7 | 77.5±1.0 | 86.3±0.6 |
| Au10 - A | 14.2±3.7 | 5.4±1.1 | 29.9±4.2 | 82.6±0.6 | 57.9±1.3 |

**Table S5.** Bacterial growths of Gram positive and Gram negative reference bacteria when incubated with 200 µg/mL of extract. Data are expressed as percentage values (means ± SD) relative to the positive growth controls (bacteria grown in regular medium).

| *Label - Season* | *Vero cells* |
| --- | --- |
| Au1 - Sp | 50.5±6.1 |
| Au2 – W_FR_ | 43.0±5.2 |
| Au2 - Sp | 40.1±1.3 |
| Au2 - A | 21.4±1.7 |
| Au4 - Sp | 44.5±2.6 |
| Au4 - S | 53.0±9.2 |
| Au4 - A | 33.6±5.1 |
| Au5 - W_FR_ | 35.1±1.3 |
| Au5 - Sp | 56.4±6.1 |
| Au5 - S | 42.7±11.5 |
| Au6 - W_FL_ | 56.4±7.2 |
| Au6 - S | 40.8±7.3 |
| Au8 - S | 29.7±3.2 |
| Au8 - A | 28.6±10.0 |
| Au9 - W_FL_ | 48.5±5.2 |
| Au10 - A | 43.8±9.0 |

**Table S6.** Cell viability and proliferation of Vero cells following 48 h of incubation with 200 µg/mL of extract. Data are expressed as percentage values (mean ± SD) relative to untreated cells
